# Enhanced functional connectivity and volume between cognitive and reward centers of naïve rodent brain produced by pro-dopaminergic agent KB220Z

**DOI:** 10.1371/journal.pone.0174774

**Published:** 2017-04-26

**Authors:** Marcelo Febo, Kenneth Blum, Rajendra D. Badgaiyan, Pablo D. Perez, Luis M. Colon-Perez, Panayotis K. Thanos, Craig F. Ferris, Praveen Kulkarni, John Giordano, David Baron, Mark S. Gold

**Affiliations:** 1 Department of Psychiatry & McKnight Brain Institute, University of Florida College of Medicine, Gainesville, Florida, United States of America; 2 Department of Psychiatry, Wright State University, Boonshoft School of Medicine, Dayton, Ohio, United States of America; 3 Department of Holistic Medicine, National Institute for Holistic Addiction Studies, North Miami Beach, Florida, United States of America; 4 Division of Applied Clinical Research & Education, Dominion Diagnostics, LLC, North Kingstown, Rhode Island, United States of America; 5 Department of Psychiatry, Keck Medicine University of Southern California, Los Angeles, California, United States of America; 6 Research Institute on Addictions, University at Buffalo, Buffalo, New York, United States of America; 7 Center for Translational Neuroimaging, Department of Psychology and Pharmaceutical Sciences, Northeastern University, Boston, Massachusetts, United States of America; Istituto Italiano di Tecnologia, ITALY

## Abstract

Dopaminergic reward dysfunction in addictive behaviors is well supported in the literature. There is evidence that alterations in synchronous neural activity between brain regions subserving reward and various cognitive functions may significantly contribute to substance-related disorders. This study presents the first evidence showing that a pro-dopaminergic nutraceutical (KB220Z) significantly enhances, above placebo, functional connectivity between reward and cognitive brain areas in the rat. These include the nucleus accumbens, anterior cingulate gyrus, anterior thalamic nuclei, hippocampus, prelimbic and infralimbic loci. Significant functional connectivity, increased brain connectivity volume recruitment (potentially neuroplasticity), and dopaminergic functionality were found across the brain reward circuitry. Increases in functional connectivity were specific to these regions and were not broadly distributed across the brain. While these initial findings have been observed in drug naïve rodents, this robust, yet selective response implies clinical relevance for addicted individuals at risk for relapse, who show reductions in functional connectivity after protracted withdrawal. Future studies will evaluate KB220Z in animal models of addiction.

## Introduction

Addiction to psychoactive drugs poses a significant threat to the health, social and economic fabric of families, communities, and nations. The number of substance users is staggering. The annual U.S. National Survey on Drug Use and Health (NSDUH) estimated that in 2013 about 24.6 million Americans aged 12 or older used illicit drugs in the past month [1]. This problem urgently requires the development novel treatments for addiction and advanced methods to evaluate the efficacy of potential therapeutic agents. Developing treatments based on well-known biosynthetic pathways that regulate central dopamine systems involved in mediating rewarding experiences is a major challenge. To curtail psychoactive drug abuse and dependence the U.S. Food and Drug Administration (FDA) approved several pharmaceutical agents collectively known as Medication Assisted Treatment (MAT) see S1 Table [2]. While these agents have helped a portion of patients over the years, they have not fully prevented drug craving and relapse. The limited success of treating psychoactive substance abuse with current modalities leaves open the need to develop new therapies [3–6].

Individuals suffering from a substance use disorder, are consequently affected by Reward Deficiency Syndrome (RDS), which is characterized by a hypodopaminergic state or trait. A hypodopaminergic state may result from the toxic effects of binge drug intake, or chronic uncontrollable stress and predisposes individuals to self-medicate to elevate or stimulate dopamine release. Normalizing dopamine, particularly in regions that comprise the reward circuitry, is one promising strategy that is consistent with recent animal models of dependence [7], and with previous theories about the role of dopamine in drug seeking and addiction [8]. Due to immediate and prolonged cellular and intracellular adaptations (e.g., desensitization and supersensitivity), pharmacotherapeutics acting selectively on postsynaptic dopamine receptors fail to normalize dopamine at a neural circuitry level. Known D2 agonists like bromocriptine [9] as well as L-Dopa [10] when administered chronically induce a down-regulation of D2 receptors, and as such, result in further unwanted dopamine dysregulation thus limiting their therapeutic usefulness. Ultimately, an important goal is to activate mesolimbic D_2_ receptors with dopamine agonist therapy capable of supplementing biochemical mechanisms mediating synthesis, control, and release of dopamine, particularly at D_2_ target sites. The nutraceutical KB220Z was assembled based on the cascade of neurotransmission that results in reward. The brain reward cascade involves the enzymatic synthesis in midbrain dopamine neurons controlled by hypothalamic serotonergic, enkephalinergic and GABAergic neurons, as well as, their targets in the nucleus accumbens (NAc) that contain high levels of D_2_ receptors [9]. Preclinical studies and human trials involving KB220 variants have been previously reviewed [9]. Prior KB220 variants have enhanced brain enkephalin levels in rodents; reduce alcohol-seeking behavior in C57/BL mice; by pharmacogenetical convertion of ethanol-preferring C57/BL mice to the same level of non-preference as DBA mice. In humans, KB220Z has been reported to reduce drug and alcohol withdrawal symptomatology [9]. A pilot study of abstinent heroin addicts found that compared to placebo a single dose of KB220Z resulted in improved prefrontal-cerebellar-occipital neural network connectivity and NAc activation [10]. Thus, based on these and other studies, the use of KB220Z might be an ideal strategy to treat RDS, particularly to counteract underlying brain hypodopaminergia.

A major limitation to furthering the advancement of novel therapeutics, such as KB220Z, has been the lack of appropriate methods for determining their effects on the *in vivo* functional organization of the Central Nervous System (CNS). The integration of brain *regions* into transient, and sometimes persistent, *functional networks* seems to be part of the brains organizational principles and may be measured by resting state functional magnetic resonance imaging (rsfMRI) [11]. There is growing support for the use of rsfMRI and functional connectivity analysis as a novel biomarker for addictive disorders. Resting state functional connectivity (rsFC) between cortical and subcortical limbic regions of volunteers dependent on alcohol, cocaine, cannabis or heroin varies from that observed in healthy volunteers [12], and in cocaine users altered rsFC correlates with poor performance on cognitive tasks [13–16]. Additional work showed clearly defined cocaine withdrawal dependent reductions in rsFC between five mesocorticolimbic ‘seed’ regions [14]. Similarly, other human fMRI work has identified a range of functional network adaptations in heroin [17–19] and alcohol users [13, 20, 21]. The utility of rsfMRI for defining functional connectivity changes in response to medications, such as atomoxetine [22], methylphenidate [23] and levodopa [24] has also been supported. Such studies greatly benefit from the translational capabilities of high field imaging in which test compounds can be first assessed in animal models using similar acquisition and analysis tools. Indeed, rodent models of disease and the use of imaging technologies provide cross-species links to understanding both the intrinsic functional organization of the CNS and for testing compounds that can alter brain connectivity patterns [25–27]. In the present study, fMRI at 11.1 Tesla was used to test the network-level actions of KB220Z. Functional connectivity patterns and connectivity volume between several brain structures including areas of the reward system in the rat were examined. The placebo-controlled experiments were designed to test whether the observed rsFC is altered in the rat brain by administration of a putative dopaminergic agonist, KB220Z.

## Materials and methods

### Subjects

Ten male Long Evans rats (350–400 grams) were obtained from Charles River Laboratories (Wilmington, MA) and housed in pairs in a temperature and humidity-controlled room (12 hr light cycle with lights off at 1900 hr). Water and Purina rat chow were provided *ad libitum*. Rats were acquired and cared for in accordance with the guidelines published in the Guide for the Care and Use of Laboratory Animals 8^th^ Edition [28] and the guidelines of the National Institutes of Health and the American Association for Laboratory Animal Science. The Institutional Animal Care and Use Committee at the University of Florida provided prior approval of the protocols used in this study.

### Composition of and preparation of KB220Z and placebo

The most recent variant of KB220Z (powdered form) used in the present study is comprised of the following ingredients: Thiamine, 15 mg (1033% of Daily Value); Vitamin B6, 10 mg (500%); Chromium poly nicotinate 200 mcg (166%) and a fixed dose of Synaptose. Synaptose is a combination of amino acids and herbs that contains DL-Phenylalanine, L-Tyrosine, Passion Flower Extract; a Complex containing Arabinogalactans, N-Acetylglucosamine, Astragalus, Aloe Vera, Frankincense Resin, White Pine Bark Extract, and Spirulina; Rhodiola; L-Glutamine; 5-Hydroxytryptophan (5-HTP); Thiamine Hydrochloride; Pyroxidal-5-phosphate and Pyridoxine HCl [9]. The powder was manufactured by Cephram, Inc. (New Jersey). Fresh solutions were prepared in double distilled water prior to imaging and delivered at a total concentration 33 mg/ml (based on weighed powder) and delivered in a total volume of 0.1ml over 30 seconds.

### High field functional magnetic resonance imaging, processing, and analysis

Animals were fitted with an oral cannula used to administer double distilled water as placebo or KB220Z (oral concentration of 33mg/ml estimated based on preliminary studies and comparable to effective doses used previously in human subjects). Preliminary studies were conducted to secure the fluid delivery cannula with its tip deep in the lateral and proximal lingual area. This oral region contains a high density of capillaries that facilitates absorption of KB220Z ingredients. The faster sublingual absorption through oral capillaries was preferred to the slower gastric delivery, which would lead to first pass hepatic metabolism and partial degradation of KB220Z ingredients. Each animal served as its own control using a crossover design with scans one week apart. Functional scans lasted 20 minutes; with a 5-minute baseline scan and following delivery of either a placebo or KB220Z a contiguous 15-minute scan. Anatomical scans lasted 6 minutes. Each rat was imaged twice, resulting in 20 scans. Of these scans, 5 were discarded due to excess motion artifact (S3 Fig), leaving 15 scans (n = 7 for placebo and n = 8 scans for KB220Z).

**Fig 1:**
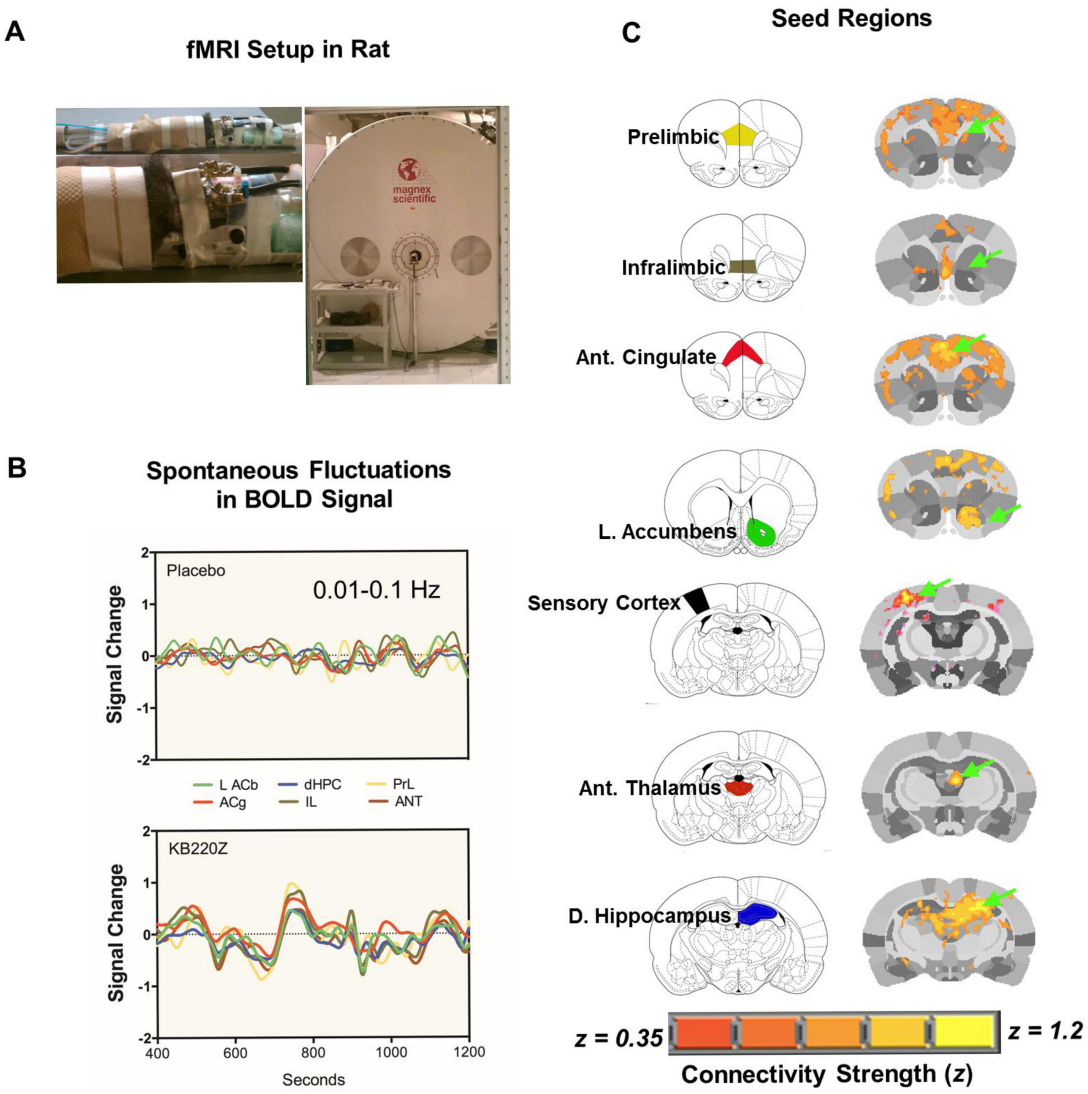
The experimental setup used for data acquisition and analysis of rat rsfMRI. (**A**) Experimental setup for fMRI data acquisition on a 470-MHz MRI using a ^1^H transmit/receive head surface coil. (**B**) Processed Blood-Oxygen-level Dependent (BOLD) signals from regions of the reward system. After skull stripping, atlas registration, motion and drift correction, and intensity normalization, images were band-pass filtered between 0.01–0.1Hz. (**C**) Anatomical location of selected seed regions within the reward system that were used for cross-correlation analysis (to generate voxel-wise maps of Pearson’s r coefficient that was later z-transformed prior to group comparisons). Shown are both a standard anatomical atlas and high-resolution MRI-based atlas maps. Green arrows highlight the seed region. Overlays on the rat brain atlas show connectivity with corresponding seed regions based on Fisher’s z-transformed r coefficient values (r ≥ 0.35) [29].

Functional MRI datasets were collected on a ^1^H 470.7MHz (11.1 Tesla) MRI scanner (Magnex Scientific) with high-performance gradients (Resonance Research Inc.; Gmax = 1500 mT/m at 150A and 130 us risetime), and controlled by a VnmrJ 3.1 console (Agilent, Palo Alto, CA). A quadrature transceive ^1^H surface coil with uniform B_1_ coverage for most of the rat brain (2.5 x 3.5cm) was used for radiofrequency signal transmission and detection Fig 1A.

Anatomical scans used for functional image overlays and subject-to-atlas alignment were first collected using a fast spin echo sequence with the following parameters: repetition time (TR) = 2 sec, echo time (TE) = 45ms, echo train length = 8, field of view (FOV) = 25.6 mm^2^, data matrix size = 256 x 256, 10 contiguous slices at 1 mm each, and 10 averages. A single shot spin echo—echo planar imaging (EPI) scan was used for the acquisition of fMRI images (TR = 4 sec; TE = 20ms; 25.6 mm^2^ x 10 mm field of view; 1 mm contiguous slices (no slice gaps); 64 x 64 data matrix). In order to minimize spontaneous alterations in rsFC, the animals were scanned under 1.0–1.5% isoflurane anesthesia. The anesthesia was delivered in a medical grade gas mixture of 70% N_2_/30% O_2_ mixture at 0.1L/min. Respiration was closely recorded and assessed (all animals showed 50–70 breaths per minute), and body temperatures were controlled by a warm water recirculation system and kept at 37°C. High levels of isoflurane (>2.8%) have been shown to disrupt rsFC. However, there is growing support that basal networks are active while under the levels of anesthesia used here [30–33]. The same 11.1 T system and similar acquisition parameters have previously shown robust drug-evoked Blood-Oxygen-level Dependent (BOLD) responses [34].

Brain masks were manually drawn over anatomical scans using the drawing tool in itkSNAP (www.itksnap.org). The masks were then used to remove the non-brain signal from each anatomical scan. The resulting cropped images were aligned with a rat brain template using the FMRIB Software Library automated linear registration tool flirt [35]. (In flirt, 12 parameter affine-registration with a correlation ratio search cost, full angular search along x y z, a mutual information cost function, and nearest neighbor interpolation, and other default parameters were used). The quality of registration was judged to be adequate for both anatomical and functional scans based on the precise alignment of important landmarks inside the brain such as the corpus callosum, lateral ventricles, hippocampus, and these were qualitatively evaluated for each subject. It should be noted that in functional scans slight misalignments are commonly observed in temporal cortical and the most rostral prefrontal areas near sinuses (a representative set of raw EPI and fast spin echo images is shown in S1 Fig). Transformation matrices resulting from anatomical-to-atlas registration were used to register functional datasets into atlas space for subsequent preprocessing and analysis steps (using commands from FSL flirt). Functional images were corrected for motion, and slice timing delays, and time series DC spikes were removed using Analysis of Functional NeuroImages (AFNI) [36]. Linear and quadratic detrending, spatial blurring, and intensity normalization were also performed. Six head motion parameters, cerebroventricular and white matter signals were removed from all datasets. A voxelwise temporal band-pass filter (between 0.01 Hz and 0.1 Hz) was applied to remove brain signals that may contain cardiac and respiratory frequencies. Regions of interest were selected within each hemisphere and analyzed separately to determine cross-hemispheric symmetrical patterns of connectivity, and to avoid averaging signals from distant seeds, like for example, left and right dorsal hippocampus. These present imaging experiments were carried out at a magnetic field strength of 11.1 Tesla and at this field there is significant enhancement of signal to noise above 4.7 Tesla. However, because of its small dimension, the ‘in house’ custom-built radiofrequency coil did not provide enough B_1_ signal coverage across the rostral-to-caudal extent of the rat brain. Thus, one of the shortcomings of the present work is that important areas of the midbrain had low signal to noise and were not sampled during acquisition because of the limited coverage. This hindered our ability to measure functional connectivity with structures such as the ventral tegmental area (VTA), interpeduncular nucleus, locus coeruleus and others that are localized in hindbrain areas. In future experiments, we expect to improve signal coverage by using a 4-channel phased array coil system for rat (Rapid MRI, Columbus, OH). It will be critical to examine the VTA since this is the main source of dopamine inputs to the nucleus accumbens.

Regions of interest were selected within each hemisphere and analyzed separately to determine cross-hemispheric symmetrical patterns of connectivity, and to avoid averaging signals from distant seeds, like for example, left and right dorsal hippocampus. Individual seed regions of interest (ROI) were chosen from the brain reward system based on rat brain atlas. These regions included the NAc, anterior cingulate cortex, dorsal hippocampus, amygdala, lateral hypothalamus and mediodorsal thalamus, see Fig 1C. From these individual time series, signals were extracted and used for correlating with the rest of the brain on a voxel-by-voxel basis using Analysis of Functional NeuroImages AFNI (http://afni.nimh.nih.gov/afni/) [36], see Fig 1B. The first 5-minutes of baseline scan were not utilized in the cross-correlation procedure. Fisher’s z-transformed images were group-analyzed using a t-test. AFNI’s 3dClustSim program was used to determine an adequate voxel cluster size for a given p-value. The resultant voxel cluster size at p < 0.05 was used to limit the chance of noise voxels in the functional connectivity maps to below 5%. Correlation coefficient values representing functional connectivity between pairs of brain regions were exported for each seed ROI for further detailed analyses comparing KB220Z rats to controls using a t-test (corrected for multiple comparisons using the Holm-Sidak method). This initial analysis revealed that KB220Z produced increases in the spatial extent of functionally connected ROI. Consequently, (using the AFNI tool 3dROIstats) per each ROI the number of nonzero voxels surviving a z threshold of 0.3 were exported, and these results analyzed as functional connectivity volume.

## Results

### Quality assessment of fMRI studies

Motion was assessed in all scans individually and scans with excess motion were removed from the study (S3 Fig). This resulted in an n = 7 scans for placebo and n = 8 for KB220Z. The quality of anatomical-to-atlas and functional-to-atlas transformations for each subject were assessed and found to be highly precise. Representative image registration for anatomical and functional scans is shown in S1 Fig. Functional connectivity between the left and right ventral and dorsal striatum were assessed to determine the presence of functional homotopic connectivity in placebo control rats, S2 Fig. Homotopic connectivity in the striatum, as previously reported, and in the NAc, was confirmed, S2 Fig. The presence of movement in images was also examined, and animals with excess motion artifact were removed shown in S3 Fig. Measured respiratory rates and body temperatures remained stable before and after administering KB220Z, with breathing typically between 50–70 breaths per minute.

### Effects of KB220Z on functional connectivity

**Fig 2:**
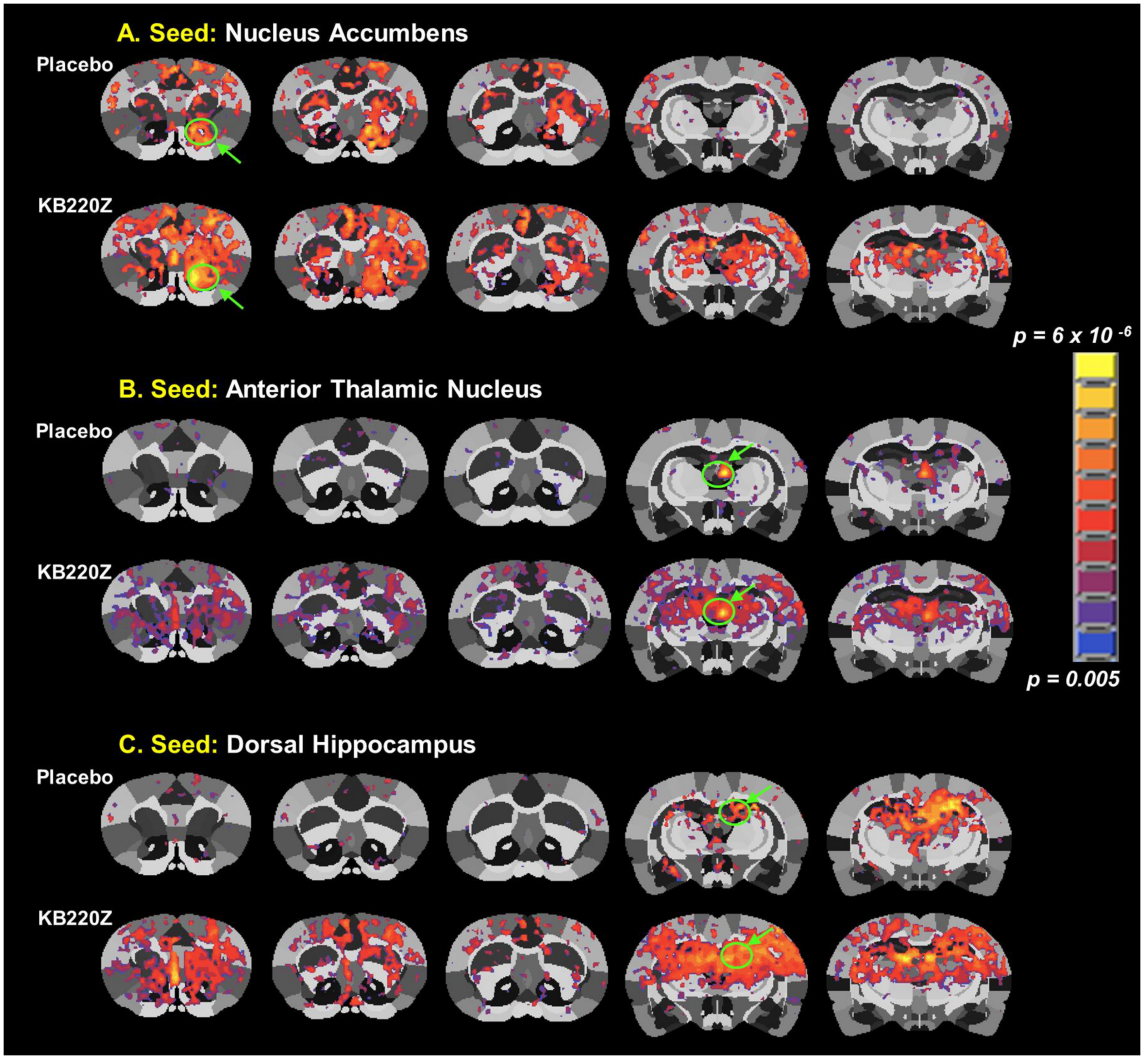
KB220Z increases functional connectivity in the rat brain reward system seed regions. Composite resting state functional connectivity maps for placebo and KB220Z for seed ROI placed on (**A**) the accumbens, only left seed is shown (n = 7 placebo and n = 8 KB220Z). (**B**) The anterior thalamic nucleus and (**C**) the dorsal hippocampus. Green arrows and circles indicate highly correlated voxels within the seed region itself. Connectivity maps are set at a lower statistical threshold of t = 4, p < 0.005 (cluster size corrected).

**Fig 3:**
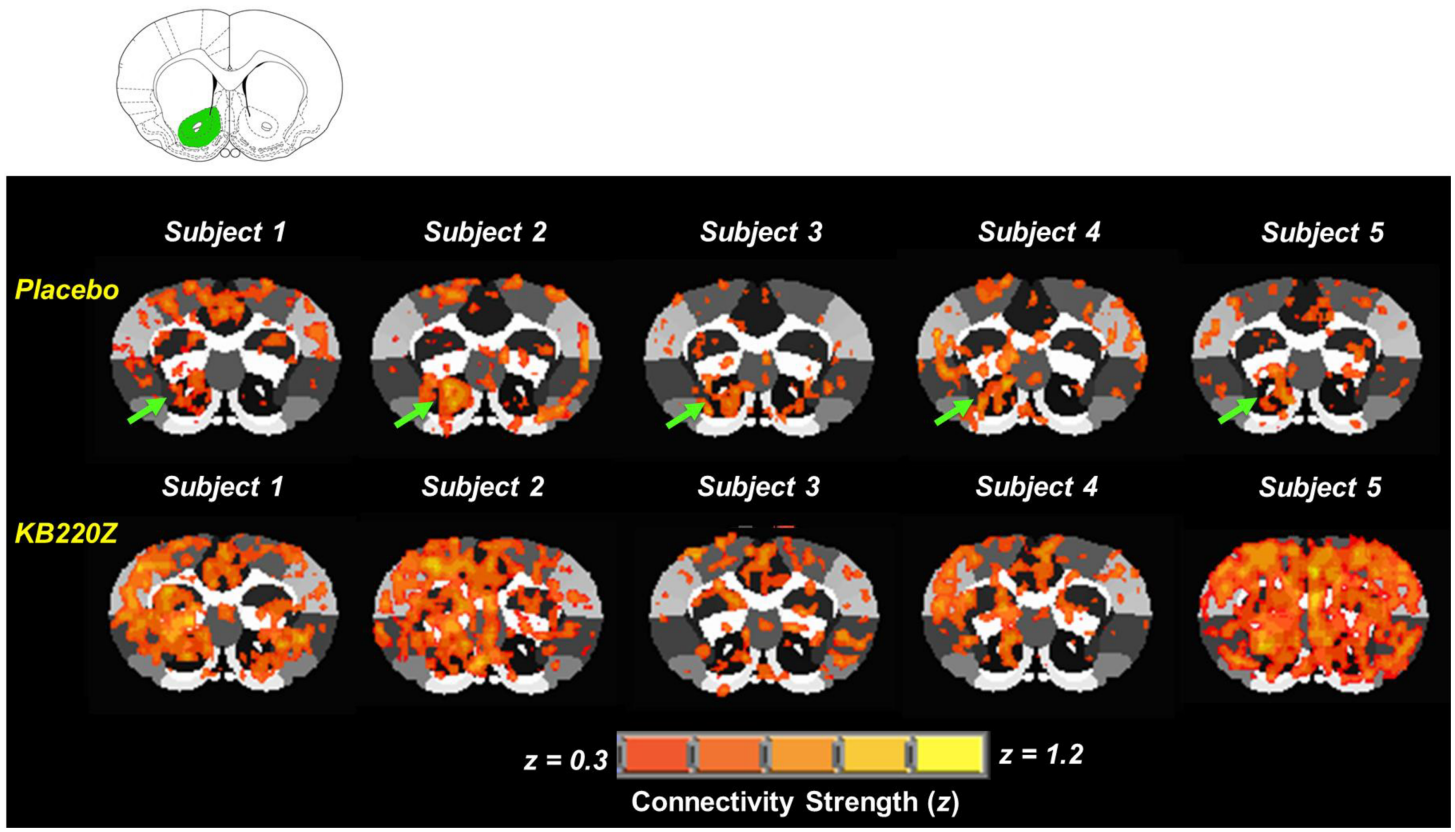
Representative cross-correlation maps show five subjects: Placebo compared to KB220Z treated rats. The maps correspond to resting state connectivity for the NAc (highlighted in green in the atlas map above the figure; only left seed is shown). Note the distributed but significant connectivity between various brain regions and the NAc in the placebo subjects. KB220Z increased connectivity, especially between left-right accumbens, dorsal striatum, and limbic cortical areas such as the anterior cingulate, prelimbic and infralimbic regions. Correlation maps for representative subjects presented at a threshold between 0.3 ≤ z ≥ 1.2.

Spontaneous oscillatory BOLD activity within the NAc was observed to correlate with the NAc sub-regions in the right hemisphere and with other regions of the brain. In Fig 2A orally administered KB220Z (33mg/ml delivered in 0.1ml, p.o.) significantly enhanced functional connectivity between the NAc and brain regions including the dorsal striatum, primary and secondary motor cortices, anterior cingulate, prelimbic and infralimbic cortices. Enhanced functional connectivity was also observed in images of individual rats Fig 3 and was not related to the presence of gross motor artifacts that would add an artificial correlative structure to the results. Importantly, increases in functional connectivity appeared specific to the regions shown in Fig 1C and not broadly diffuse across all brain regions.

There was a significant increase in functional connectivity of NAc with medial and lateral anterior thalamic nucleus and surrounding somatosensory cortex, Fig 2A. Thus, it appears that treating rats with KB220Z increased connectivity between corticothalamic areas and this region of the reward system. The treatment also appears to have resulted in the recruitment of additional brain regions outside the reward system, potentially including these within an integrated network. The reason for this recruitment is unclear, but likely leads to the emergence of behavioral features in association with the respective functions of the individual structures. Furthermore, when the anterior thalamic nucleus was the selected seed region evidence of connectivity observed outside of this area, was minimal with placebo, Fig 2B. However, there was a significant increase in connectivity with surrounding sensory cortical areas, and the regions mentioned above, including NAc (both left and right) with KB220Z. An even stronger effect on rsFC was observed in the dorsal hippocampus, see Fig 2C. No evidence of significant functional connectivity outside of the seed region was found in the placebo control scans while a dramatic and significant increase in connectivity was observed with KB220Z. Connectivity was enhanced between the left and right dorsal hippocampi, the surrounding barrel field and upper limb somatosensory regions, the NAc and limbic cortical areas including the anterior cingulate.

Implicit in the above-summarized results is the selectivity of the effects of KB220Z on the directionality of connectivity between three ROI involved in processing reward and memory. When the NAc of KB220Z -treated animals was selected as the seed region the averaged time series used for pair-wise cross-correlation analysis included harmonics also present in the hippocampus and anterior thalamic nucleus. The untreated control animals, by comparison, demonstrated very little if any interactions between these regions. A similar effect was described for the latter two regions (dorsal hippocampus and anterior thalamic nucleus) in KB220Z -treated animals. In the placebo control scans, there was a noticeable lack of significant correlations with rsFC in the NAc.

**Fig 4:**
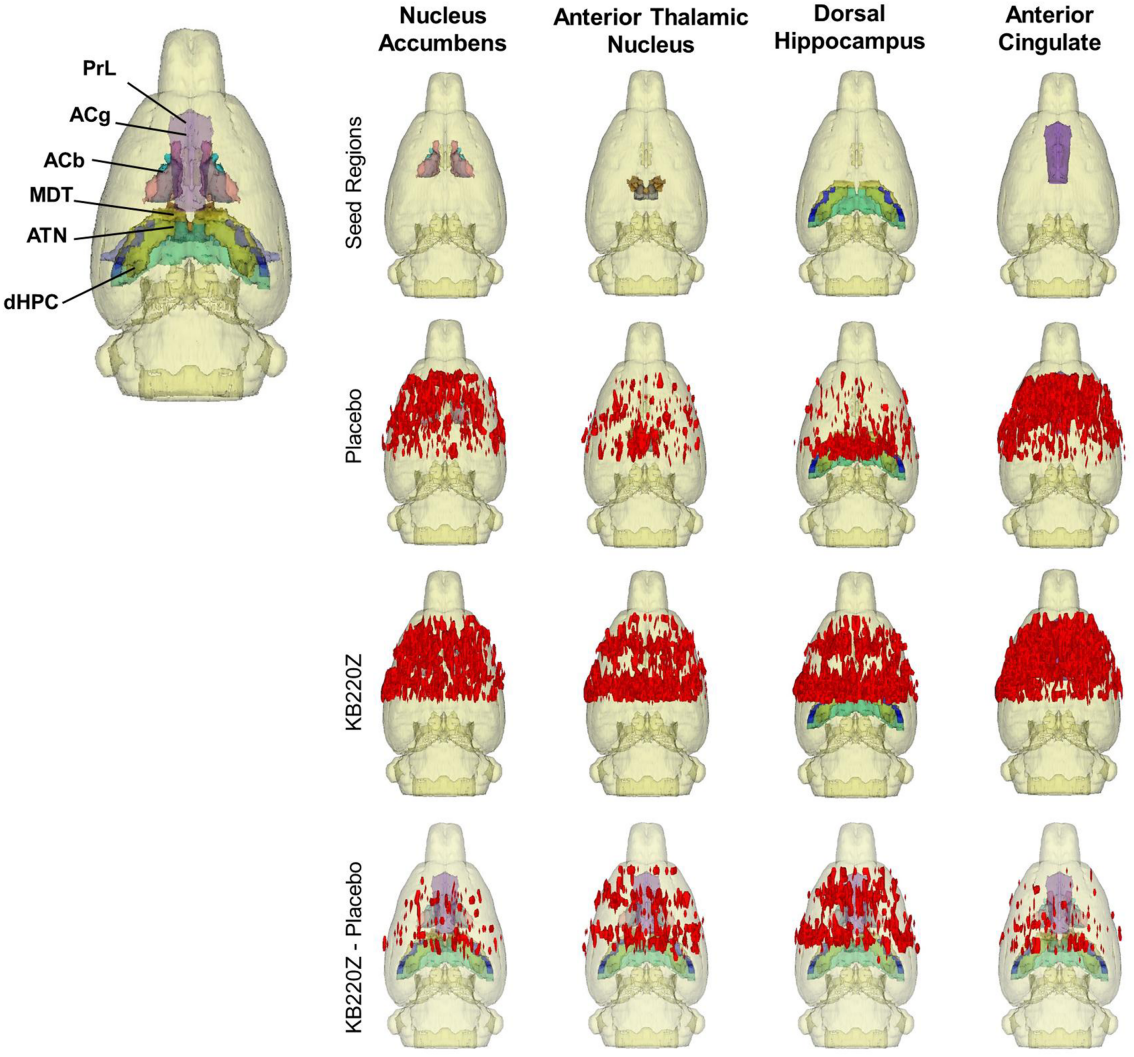
Composite three-dimensional functional connectivity maps comparing KB220Z and placebo. The top row shows the segmented 3D ROI used as seed for the placebo and KB220Z maps seen below them. High clustering of voxels occurs within the seed regions for both placebo and KB220Z groups. Greater connectivity based on the number of voxels showing high correlation coefficient values is observed in the KB220Z maps. Difference maps (KB220Z minus placebo) are shown in the bottom row. Maps are set at a lower statistical threshold of p < 0.005 (voxel cluster size corrected).

In addition to partly demonstrating a network-level action of KB220Z on rsFC, Fig 4 illustrates a significant increase in the volume of tissue activated by the treatment. In other words, rather than only increasing the degree of correlation between different brain regions, a greater volume of correlated voxel-wise signal changes in the examined brain structures was also observed. The 3D distribution of brain voxels with highly correlated spontaneous BOLD signal fluctuations were analyzed and confirmed, Fig 4. The 3D composite maps further support a greater functional connectivity within the reward system and other associated structures and the recruitment of various structures by KB220Z.

**Fig 5:**
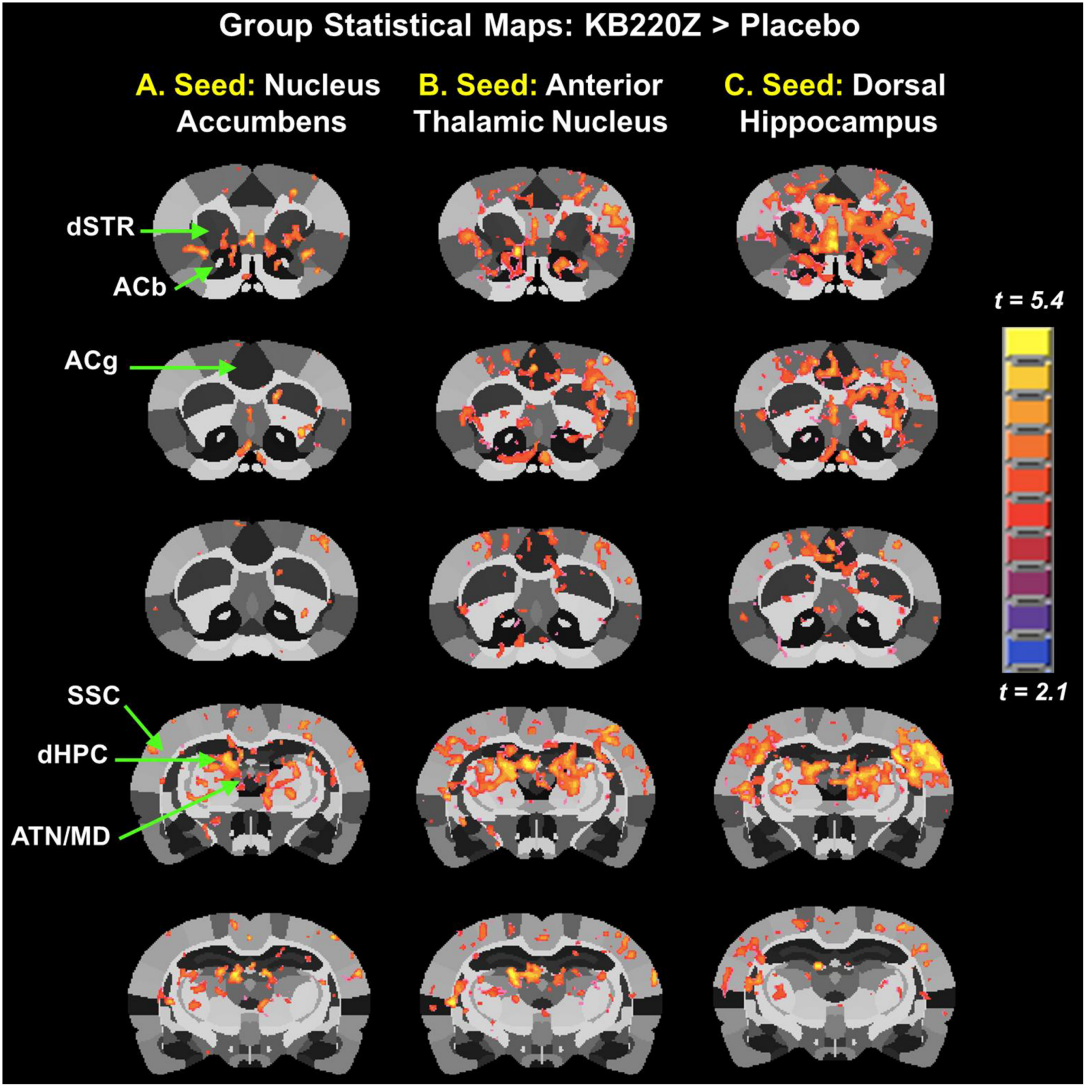
Group statistical maps comparing KB220Z and placebo treatments. Data are shown for three different seed regions: (**A**) accumbens (ACb), (**B**) anterior thalamic nucleus (ATN) and (**C**) dorsal hippocampus (dHPC), only left representations are shown here. Voxel-wise statistical t-tests (KB220Z vs. Placebo, p < 0.05, two tailed, heteroscedastic variances; cluster-size corrected using 3dClustSim on AFNI were used to compare the two groups. Other ROI indicated by green arrows are the dorsal striatum (dSTR), anterior cingulate (ACg), accumbens (ACb), somatosensory cortex (SSC); and the mediodorsal thalamus (ATN/MD).

To examine and confirm that the specific regional differences between placebo and KB220Z were significantly different, we carried out a t-test between the two groups. Group statistical maps (Fig 5) show the voxels that demonstrated a greater correlation of spontaneous BOLD fluctuations with KB220Z than with placebo treatment (p < 0.05, cluster size corrected). Once again increased connectivity between the left and right NAc was seen and increased connectivity with thalamus and dorsal hippocampus was observed with KB220Z treatment.

**Fig 6:**
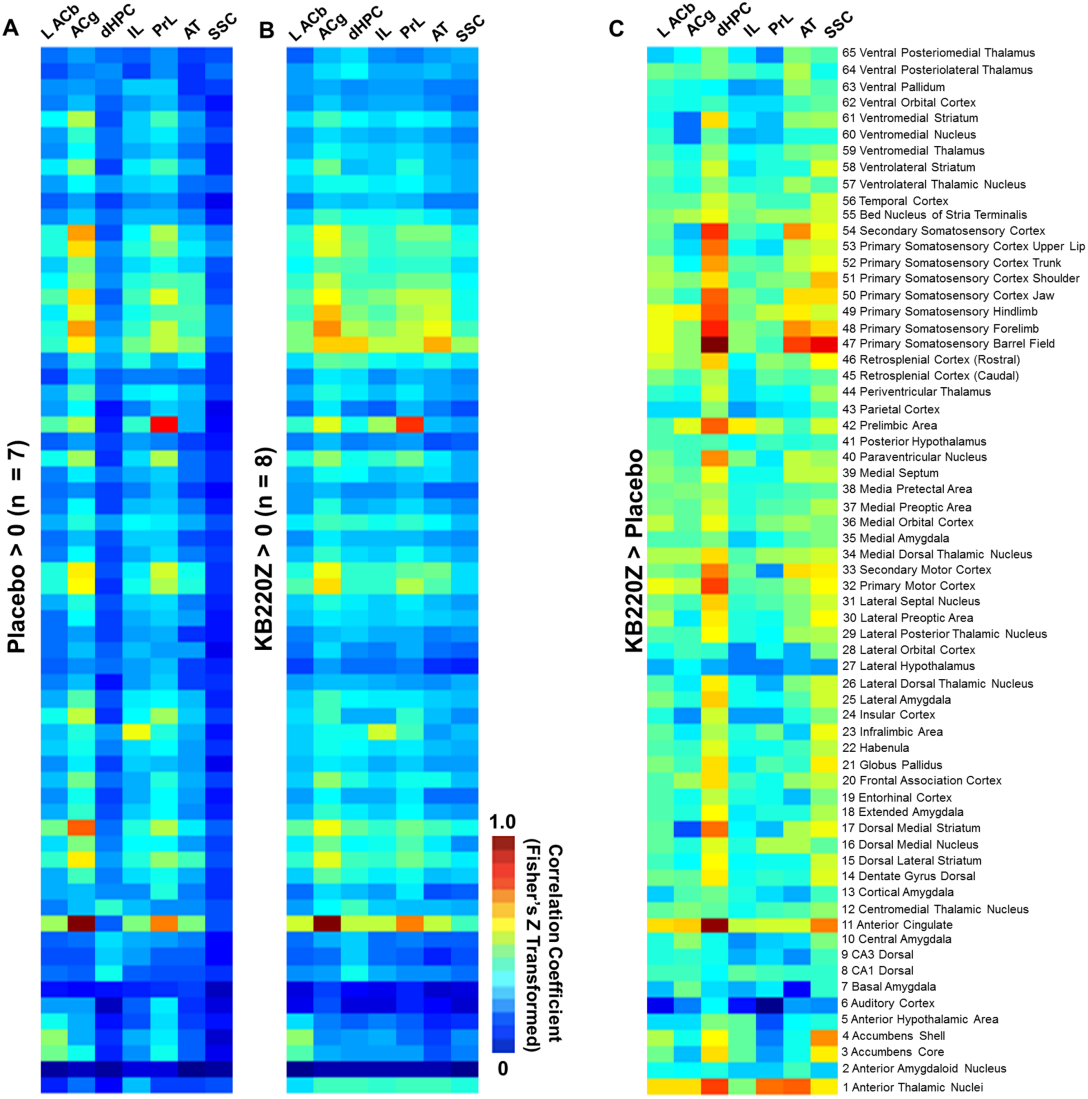
Heat maps of cross-correlation coefficient values (Fisher’s z-transformed) for seed regions and 65 rat brain atlas ROI. (**A**) Data summary for placebo treatment. (**B**) Data summary for KB220Z treatment. (**C**) Difference map of correlation coefficient values greater in KB220Z than placebo. The scale bar color indicates degree of connectivity (cool-blue < warm-yellow < hot-orange/red). Note the overall similar pattern of connectivity within and outside brain reward regions between A and B. The degree of connectivity is strengthened with KB220Z treatment compared to placebo (B vs. A) and is observed in the difference heat map in (**C)**. ROI that correspond to each row in the color-coded maps are shown on the far left. The seed region corresponding to each column are left accumbens (L ACb), anterior cingulate cortex (ACg), dorsal hippocampus (dHPC), infralimbic area (IL), prelimbic area (PrL), anterior thalamus (AT) and somatosensory cortex (SSC).

**Fig 7:**
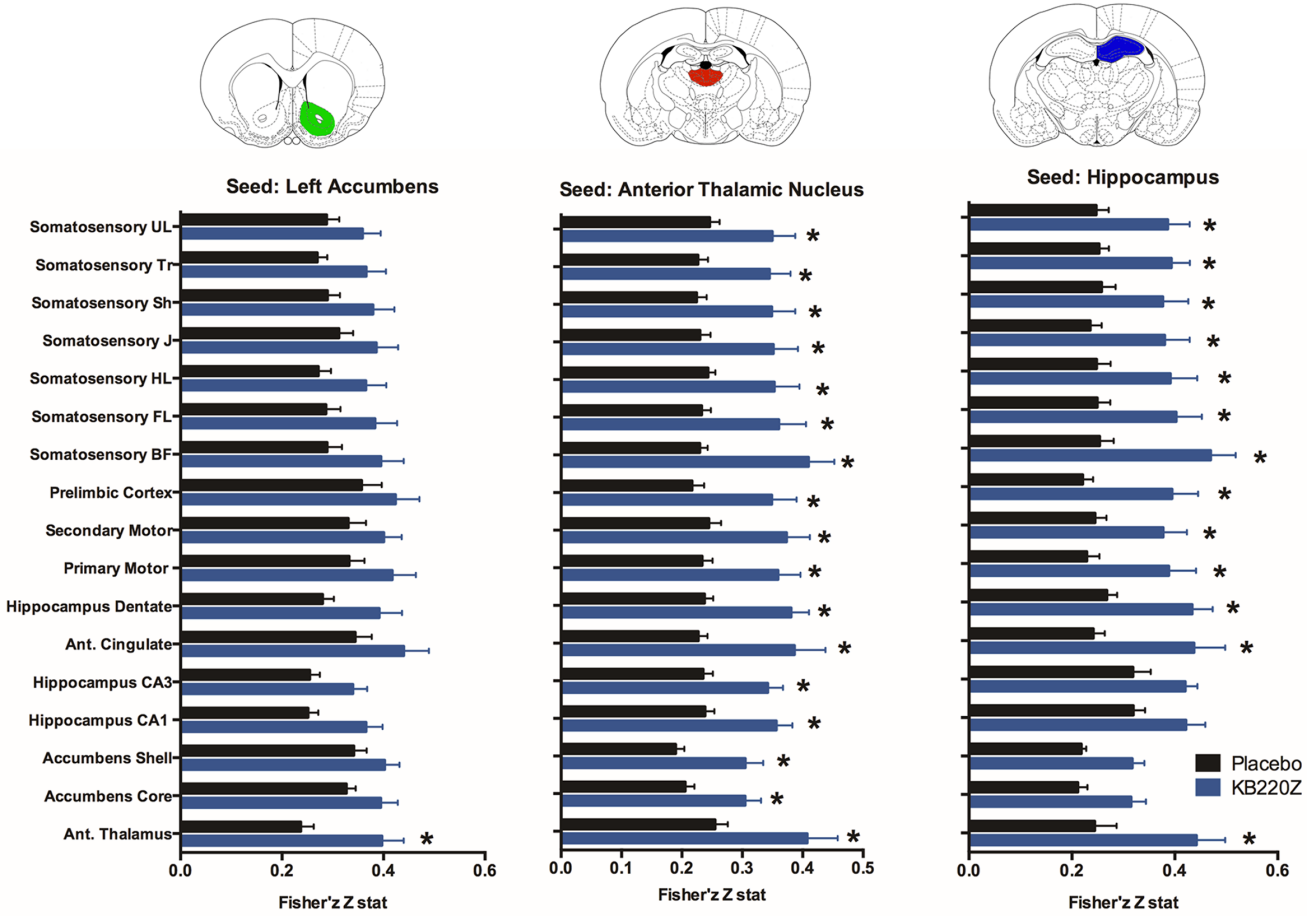
Resting state functional connectivity is higher with KB220Z than with placebo. Plots show differences in functional connectivity between various ROI and three seed regions. Data are presented as mean correlation (z transformed) ± standard error. * Significantly different than placebo t_221_ > 2.1 p < 0.05 (p values FDR corrected at q = 0.05).

The heat maps shown in Fig 6 summarize the cross-correlation results between seven different seed regions with 65 other ROI. The use of the atlas permitted segmentation of correlation coefficients from a larger series of structures within the rat brain and a detailed analysis of the potential functional interactions was performed. The overall pattern of connectivity that arises from visual observation of the color-coded coefficient values in these heat maps, illustrates a baseline state that is similar and reproducible between the crossover design scanning sessions, Fig 6A and 6B. However, a greater intensity in the degree of correlation is observed within select ROI with KB220Z treatment compared to placebo Fig 6C. The regions included were somatosensory cortex, anterior cingulate and prelimbic area, and anterior thalamic nuclei. A quantitative summary of the results for several regions is provided in the bar plots in Fig 7.

**Fig 8:**
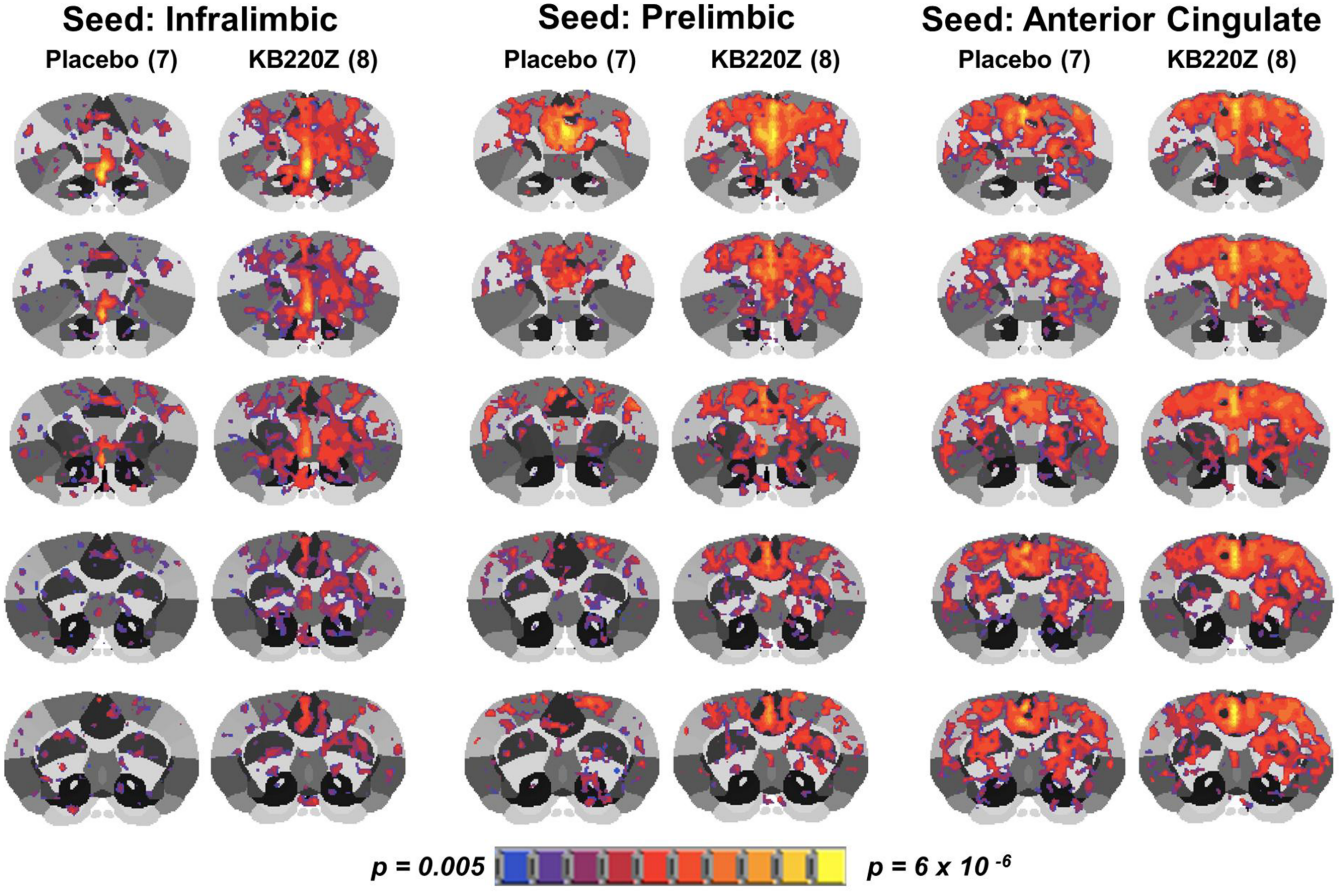
KB220Z effects resting state functional connectivity within the limbic cortex. Mean connectivity maps are at a threshold z value of 0.35 for visualization of resting state networks (scale bar indicates connectivity strength based on z value).

The intrinsic rsFC of prefrontal-limbic-cortical areas were examined. The selected seed regions have a substantial role to play in emotional reactivity, relapse, and cognitive functioning. Within the seed regions of the infralimbic, prelimbic and anterior cingulate areas, KB220Z was observed to increase the degree and extent of connectivity with surrounding cortical areas and other subcortical structures, which included striatal regions, Fig 8. However, while a qualitative difference is apparent, differences between placebo and KB220Z did not reach statistical significance. Thus, the effects of KB220Z on functional connectivity appear to be specific to NAc, anterior thalamic and hippocampal areas (Figs 5–7).

**Fig 9:**
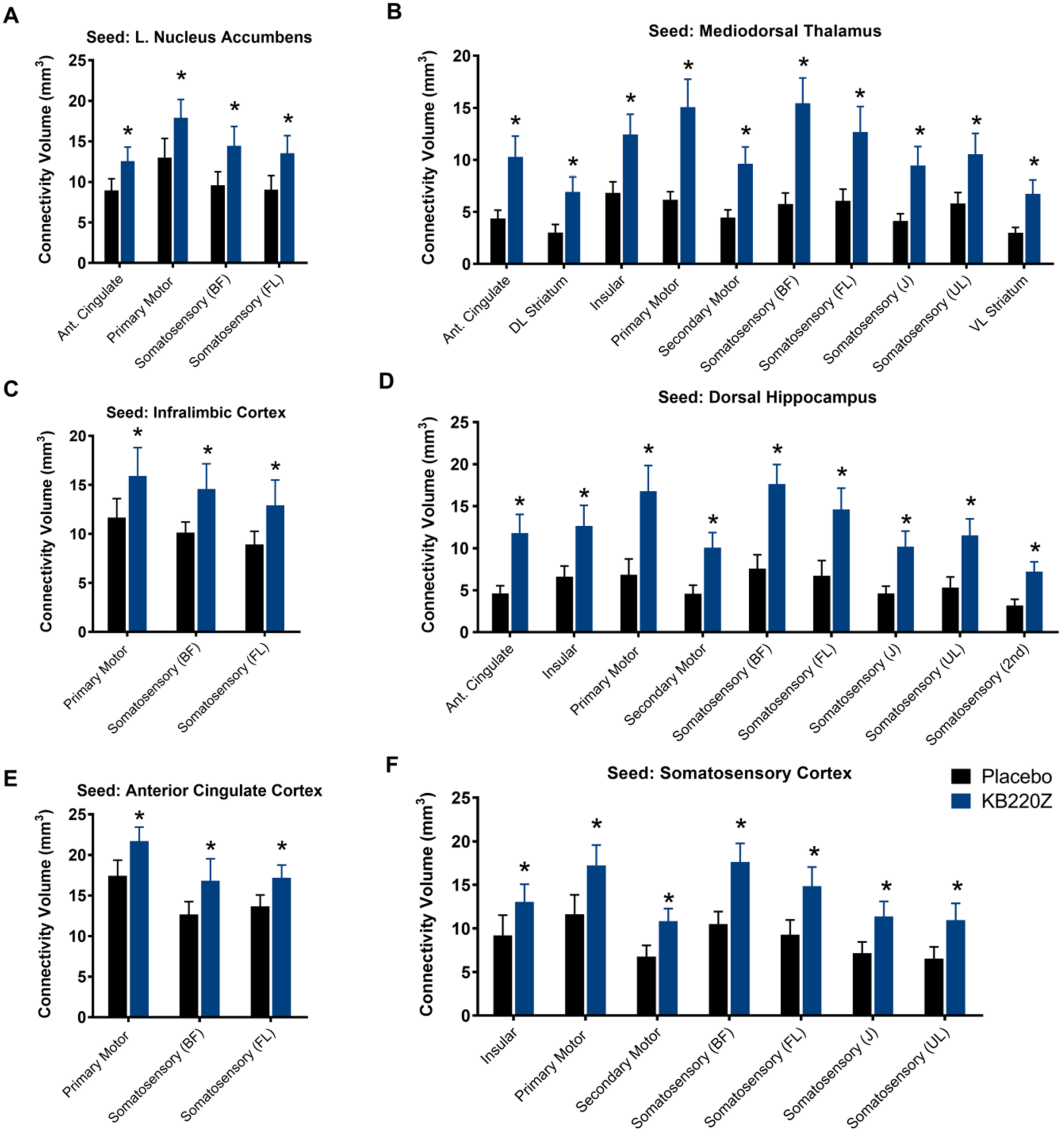
KB220Z increased the volume of connectivity. Connectivity volume was assessed by applying a correlation threshold value of z = 0.3 to all subjects and quantifying the volume above this threshold. Voxels were then converted to mm^3^ based on 3D voxel resolution. Shown are connectivity volume data for various regions including (**A**), nucleus accumbens, (**B**) mediodorsal thalamus, (**C**) infralimbic cortex, (**D**) dorsal hippocampus, (**E**) anterior cingulate cortex, and (**F**) somatosensory cortex. *Significantly different from placebo t ≥ 3.4 p ≤ 0.05 with multiple comparison corrections using the Holm-Sidak method. Sixty-five ROI were analyzed for each seed in A-F and areas of the brain with increases in functional connectivity with KB220Z treatment are shown.

Finally, the strength of connectivity, through an assessment of the correlation coefficient values in the placebo and KB220Z groups was determined. Significant differences between these two groups regarding the volume of connectivity values above a threshold value of 0.3 were noted [29]. Recruitment of more ‘neuronal territory’ within each ROI could possibly have substantial importance for enhancement of a neuroplasticity mechanism. Further, such a plasticity-like mechanism may occur as a result of enhanced dopamine function, due to the target actions of ingredients in KB220Z. Thus, the volume of connectivity per each ROI was examined, shown in Fig 9. The volume of connectivity in the ROI’s as shown in Figs 7 and 8 was assessed. Rats treated KB220Z showed greater connectivity volume than placebo controls. Interestingly, increased connectivity volume was observed between NAc and anterior cingulate (t_845_ = 3.4, p = 0.0008), mediodorsal thalamus and anterior cingulate (t_845_ = 5.6, p < 0.000001), and dorsal hippocampus and anterior cingulate (t_845_ = 6.1, p < 0.000001). We observed increased functional connectivity volume in response to KB220Z involving other brain regions, which included somatosensory and motor cortical areas (t ≥ 3.4 p ≤ 0.05).

## Discussion

It has become increasingly apparent that drug addiction involves adaptations in the genetic and epigenetic landscape of brain reward circuitry and its associated limbic and cognitive regions [37–40]. As a result of these neurogenetic adaptations, biosynthetic pathways controlling the production, storage, and release of dopamine are impaired [40–45]. Such impairments underlie RDS, which is characterized by hypodopaminergia; and this disease can be reflected in changes in functional connectivity [13–16] that could, in turn, be a manifestation of unbalanced functional circuitry [46], or due to loss of dopamine ‘homeostasis’ [47]. The pro-dopaminergic nutraceutical KB220Z contains several key amino acid precursors, enzymatic cofactors, and other ingredients assembled to increase dopamine synthesis and release in brain reward regions. KB220Z was administered to rats undergoing fMRI to determine its effects on functional connectivity, particularly between the NAc, hippocampus, mediodorsal thalamus and several prefrontal regions implicated in addiction and drug seeking. The results show that compared with placebo controls, KB220Z rats show significant increases in functional connectivity. While dopamine was not measured directly, the effects of KB220Z on functional connectivity suggest that it may increase dopamine functionality and thus result in facilitated interactions between multiple brain regions, perhaps through dopamine-mediated actions. The literature showing that subjects with substance use disorders and extended withdrawal have impairment in functional connectivity in cognitive and reward sites is growing [17, 19, 21]. The hypothesis is that KB220Z may correct these impairments at least in a sizeable proportion of the addiction population with hypodopaminergia. KB220Z may offer a means to enhance or balance functional connectivity networks reliant on intact dopamine function. In the future, this hypothesis merits further investigation, particularly in preclinical imaging experiments employing drug-seeking models.

Functional connectivity as seen in the results of the present study is consistent with previously published networks seen in studies that also used seed-based analysis [29, 32, 48, 49]. Interhemispheric functional connectivity within NAc has been previously reported in human subjects [15]. Homotopic connectivity is reported in humans and represents a potential organizational principle linking activity between hemispheric counterparts in the brain [15]. We observed a similar finding in the rat brain, and the results are presented in composite maps shown in Fig 2. Seed ROI including the nucleus accumbens, cingulate gyrus, anterior thalamic nuclei, hippocampus, prelimbic and infralimbic loci showed significant functional connectivity increases with KB220Z. This response induced by KB220Z demonstrated enhanced dopaminergic functionality across the brain reward circuitry, never previously measured increased significant functional connectivity, and brain volume recruitment (possible evidence for neuroplasticity).

One important question relates to the use of anesthetic and sedative agents. While isoflurane allows detection of resting state networks in rats, the connectivity is weaker with higher concentrations of isoflurane (for example >2.2–2.8%) [50]. Indeed, anesthesia choice represents a challenge to reproducibility across many preclinical imaging studies measuring fMRI connectivity. Interhemispheric connectivity also varies with increasing concentrations of isoflurane [51]. Some groups have shown that the effects of isoflurane are complex and may affect low and high frequency signals in opposite directions [51]. However, even with variability in the connectivity of both the experimental and control animals possibly due to the use of anesthesia, confidence in the results showing that KB220Z increases connectivity between nucleus accumbens with other brain regions remains high. Under the influence of anesthetic and sedative agents, rats show spatial patterns of non-evoked functional connectivity resembling those observed in humans and non-human primates at rest [29, 52]. This similarity applies in particular to structures of the default mode network (DMN) [53]. Since cocaine abuse impacts the functional interactions of human DMNs [54] it is possible that it may have similar effects in rats and across all mammalian species [53].

Notably, many rodent studies use low concentrations of inspired isoflurane (1–1.5%) that are, at times, combined with a continuous intramuscular infusion of the sedative agent dexmedetomidine (0.07mg/kg/hr). Recent results of these studies have shown that by itself low isoflurane (1–1.5%) does allow consistent resting state networks to be obtained. Thus 1–1.5% isoflurane which is in the low-to-mid range was used, whereas it is known that doses above 1.8% can impact results. Liang et al. report the results from a comprehensive graph-theoretical approach they used to examine the intrinsic organizational properties of the anesthetized versus the awake rat brain [55]. The analysis included 114 anatomical ROI using a previous version of the rat brain atlas [56]. The functional resting state data was submitted to graph theoretical interrogation that included regions of the reward system. Functional connectivity strength was found to be increased among a set of structures reported here that included the striatum, hippocampus, anterior thalamus. Moreover, although it is unclear whether or not increased dopamine activity in the reward system affects rsFC, there is, however, indirect evidence showing that the architecture of cortico-subcortical network connectivity is influenced by mechanisms involving dopamine [24].

### Are KB220 variants putative modulators of dopamine homeostasis?

The development of KB220Z followed the first report concerning the enkephalinase inhibitor D-phenylalanine [57]. The design of the KB220Z complex follows the brain reward cascade with the final intent of facilitating dopamine release throughout the reward circuitry. While, as yet, the actual release of dopamine has not been determined, we are planning to use a single scan dynamic molecular imaging technique to understand the nature of dopamine released in the human and animal brains following administration of KB220Z [58]. This technique allows detection, mapping, and measurement of dopamine released endogenously following a pharmacological or behavioral challenge [59, 60]. Using this technique, dopamine release was observed in some of the same areas where enhanced connectivity was seen in the current experiment [61].

The hypothesis here is that these robust and selective results are due to inhibition of gamma-aminobutyric acid (GABA) transmission in the substantia nigra through serotonergic–opioidergic–glutaminergic interactions that reduce the inhibitory control of GABA over dopamine release throughout the reward network [62]. In support of these findings, similar results were found in humans showing enhanced regulation of deregulated widespread theta in the cingulate gyrus of abstinent psychostimulant abusers. Alpha and low beta waves were increased one-hour after KB220Z administration [63]. Previously, this laboratory found resting state functional abnormalities in abstinent heroin-dependent individuals affecting functional brain organization, which could negatively impact decision-making and inhibitory control [10]. Moreover, compared to normal controls; heroin addicts showed reduced activation in right amygdala in response to the affective pictures [10], consistent with previous reports of blunted subjective experience for affective stimuli in drug abusers. Other studies showed persistent abnormalities in the brain function following one month of heroin withdrawal in the orbitofrontal cortex [64]. Zijlstra et al. found lower baseline D2R availability in opiate-dependent subjects than in the left caudate nucleus of controls [64]. After cue-exposure, opiate-dependent subjects demonstrated higher dopamine release in the right putamen than controls this release positively correlated with chronic craving and anhedonia. Dopamine 2R availability in the putamen was negatively correlated with years of opiate use [65]. Treatment strategies that increase D2Rs may be an interesting approach to prevent relapse in opiate addiction [66]. Extensive evidence indicates that current and recently abstinent cocaine abusers compared to drug-naïve controls have decreased gray matter in regions such as the anterior cingulate, lateral prefrontal and insular cortex [62]. Following optogenetic stimulation of the rat NAc, brain regions crucial for cognitive processing including; the dorsal hippocampus, anterior thalamus and regions the central striatal reward structure were also observed to have changes in metabolic activity. Small animal positron emission tomography (PET) and (18)F]2-fluoro-2-deoxy-D-glucose (FDG) [27] or presentation of cues [67] were used to observe these changes. Wang et al. have shown a dysfunction of the frontostriatal and frontocerebellar circuits in heroin addicts implicating an altered balance between local neuronal assemblies-activity and their integrated network organizational pattern that may be involved in the process of moving from voluntary to habitual and compulsive drug use [17]. In a pilot human study KB220Z demonstrated improvement in the cerebellum, the cingulate and other areas of the reward circuitry in abstinent heroin addicts [10].

Importantly, the finding of increased areas of activation could have therapeutic value, especially in light of the reduced brain gray matter volume during cocaine administration to humans as reported by Connolly et al. [68]. It is plausible that KB220Z due to COMT inhibition (via Rhodiola Rosea) could result in larger amounts of dopamine in the synapse, and for that reason enhanced dopamine activity [69].

One element of the reward circuitry, the mesolimbic dopamine system in the brain controls human responses to food, social interactions, and money, and is, therefore, an important determinant of rewards and motivation. The midbrain dopamine neurons that project to the striatum are involved in producing reward. The present study illustrates the modulatory actions of a putative dopamine agonist (KB220Z) upon rsFC in association to a key region of the reward system, the NAc. The finding is that KB220Z, like optogenetic stimulation of the rat NAc [27], increased connectivity between this central striatal reward structure and the dorsal hippocampus and anterior thalamus, areas crucial for cognition. Indeed, recent work from Ferenczi et al. [70] clearly demonstrated that midbrain dopamine neuron stimulation drives both reward-seeking behavior and striatal fMRI BOLD activity. They also showed that silencing of dopamine neurons drives avoidance behavior [71] and suppresses response in the striatum, as well as other brain regions like the hypothalamus. Also, suppression of striatal responses to dopamine and inhibition of the behavioral drive to seek out natural rewarding stimuli through dopamine neuron stimulation was observed following dopamine neuron silencing. Most importantly, they demonstrated that medial prefrontal cortex (mPFC) excitability that is consistently elevated, synchronizes corticolimbic BOLD and electrophysiological activity, which can, in turn, predict anhedonic behavior in individual animals [69]. Interestingly, mPFC has glutaminergic neuronal input, and as such, there is a need to balance and optimize the fine interaction between mPFC -Glutaminergic input to striatal midbrain dopamine so that the resultant release of dopamine at the VTA–NAc is balanced [70]. These new findings have direct implications for the important observation that KB220Z potentially may induce BOLD activation due to a glutaminergic-dopaminergic optimization.

### Dopamine modulation of resting state networks: Implications for treatment

While these are the first results to show a putative modulatory effect of KB220Z, there are recent human studies that have examined the role of dopamine in rsFC, which together with the present novel finding, may provide additional insight. Importantly, Cole et al. reported that relative to placebo, levodopa, and haloperidol challenges, respectively, increased or decreased the functional connectivity between 1) the midbrain and a DMN, 2) the right caudate and a right-lateralized frontoparietal network, and 3) the ventral striatum and a frontoinsular network. Specifically, the L-Dopa treatment resulted in increased functional connectivity in regions, which included the NAc and temporal-parietal areas, as observed in rats treated with KB220Z [24]. Cole et al. also found drug-specific associations between brain-circuitry reactivity to dopamine modulation and individual differences in trait impulsivity, showing dissociable drug-personality interaction effects across distinct dopamine-dependent cortico-subcortical networks [24]. Interestingly, administering methylphenidate (which elevates extracellular dopamine levels), to non-abstinent cocaine abusers leads to region-specific alterations in the strength of connectivity, with the striatal regions becoming less connected while cortico-cortical and cortico-limbic regions showed greater connectivity [23].

The robust finding of an enhanced connectivity volume following administration of KB220Z compared to placebo suggests neuroplasticity and has important clinical relevance in addicts showing reduced mesolimbic functional connectivity. Interestingly, Tomasi et al. reported that compared to neutral cues, food and cocaine cues increasingly engaged the cerebellum, orbitofrontal, inferior frontal, and premotor cortices and the insula and disengaged the cuneus and DMN [72]. They found that these fMRI signals were proportional to striatal D2/D3 receptors. Surprisingly cocaine and food cues also deactivated ventral striatum and hypothalamus [72]. Compared to food cues, cocaine cues produced lower activation in the insula and postcentral gyrus, and less deactivation in hypothalamus and DMN regions. Activation in cortical regions and cerebellum increased in proportion to the valence of the cues, and activation to food cues in somatosensory and orbitofrontal cortices also increased in proportion to body mass. Longer exposure to cocaine was associated with lower activation to both cues in occipital cortex and cerebellum, which could reflect the decreases in D2/D3 receptors associated with chronicity. It is noteworthy that Meng et al. showed that lesion of somatosensory cortices before, rather than after morphine conditioning impaired the acquisition of place preference suggesting the importance of this region in reward–like behavior [73]. Also of interest is that highly palatable food cues in obese versus normal weight rats showed a difference in brain glucose metabolic activity. Regions in the hippocampus (memory), insular cortex (interoception), medial thalamus and in the olfactory bulb, and the parietal, and occipital cortex were involved [27]; and this involvement was proportional to D2 receptor levels [25, 26, 29].

The enhanced connectivity volume observed herein with KB220Z is supported by earlier work in abstinent human heroin addicts [10]. KB220Z induced an increase in BOLD activation in caudate-accumbens-dopaminergic pathways compared to placebo 1-hour after acute administration. It was also found that KB220Z compared to placebo, increased functional connectivity in a putative network that included the dorsal anterior cingulate, medial frontal gyrus, nucleus accumbens, posterior cingulate, occipital cortical areas, and cerebellum. The selectivity of brain regions among the more than 65 possibilities tested in this rat study, showing connectivity volume enhanced above placebo, suggests that this connectivity is not widespread but seems to be linked to reward and cognition circuitry. Interestingly, Selemon [74] found that synaptic plasticity in the frontal lobe was modulated by dopamine D1 receptors. Gass et al. found that the D2 selective blocker Haloperidol significantly reduced functional connectivity between the substantia nigra and several brain regions, notably the cingulate and prefrontal cortices, posterodorsal hippocampus, ventral pallidum, and motor cortex [75]. Moreover, the Haloperidol-induced focal changes in functional connectivity were found to be the most strongly associated with ascending dopamine projections. Further, in support of dopamine D2 receptor involvement in enhanced connectivity volume, in the present study with KB220Z, Madularu et al. reported that chronic administration of haloperidol reduced connectivity volume in awake female rats [76].

#### Experimental caveats and future perspectives

Despite these promising initial findings concerning the effects of KB220Z on functional connectivity, several experimental areas can benefit from improvements. KB220Z’s direct effect on dopamine synthesis and release has yet to be investigated. Also, the interactions between dopamine function, resting state, and evoked fMRI responses need further exploration.

It is well known that psychostimulants and other drugs acting through dopamine and other catecholamines can perturb peripheral physiological conditions [77–79]. Administration of pro-dopaminergic agents cause peripheral autonomic effects that reduce the quality of fMRI data sets and may produce non-specific artifacts associated with perturbations in systemic physiology (e.g., altered blood pressure, blood pH, arterial O_2_ saturation) [77]. Centrally, dopamine applied to brain tissue slices with functional vascular beds can cause vasoconstriction directly through dopamine receptors on arteriolar smooth muscle cells [80]. However, KB220Z does not act through similar mechanisms and therefore is not expected to alter physiology under the conditions in which the rats were imaged here. The KB220Z formulation contains amino acid precursors for dopamine synthesis and release and is not a dopaminergic drug per se. In other words, KB220Z is not a drug that would bind peripheral dopamine or adrenergic receptors to cause severe autonomic activation expected of other drugs, such as apomorphine. For instance, L-Tyrosine administered either centrally or peripherally (chronically) at various doses to rats had no effect on blood pressure in rats [81]. DL-Phenylalanine on the other hand has been reported to slightly reduce blood pressure in humans [82]. Passiflora incarnata is antihypertensive in spontaneously hypertensive rats [83] and 5-Hydroxytryptophan reduced blood pressure in hypertensive patients [84].

Importantly, cocaine seeking, especially in extended access and withdrawal paradigms, and treatment with KB220Z needs to be investigated. With regards to the fMRI studies, experiments were conducted in sedated animals and therefore studies using awake fMRI paradigms as previously reported [85] will be considered. Isoflurane and other anesthetics can potentially interact with the compounds of interest in pharmacological fMRI studies and can thus potentially modify the neural response. There is no evidence that the ingredients comprising KB220Z can interact or modify the efficacy of isoflurane or other anesthetics, and our assessment of breathing rates and body temperature indicate that the animals were under stable conditions during the short scanning sessions.

These initial findings supported by many clinical trials of KB220 variants, S2 Table are encouraging, however, the scientific and clinical addiction treatment community must be cautioned regarding interpretation of these seemingly interesting results. The sample size is rather small, although this is a placebo–controlled crossover study. A larger study of Male Long Evans rats (300 grams), compared to, for example, Lewis and Fischer 344 rats could be informative. These rat strains have been used as a model to evaluate genetic vulnerability to drug addiction, and they differ in their dopaminergic systems and warrant study involving rsfMRI. In fact, Sánchez-Cardoso et al. reported differences in the modulation of dopaminergic receptors (D1 and D2) after morphine self-administration and during extinction between these two rat strains [86].

Follow-up experiments are planned using microdialysis and optogenetics to show potential dopamine release across the various “seed” regions of the brain following administration of KB220Z.

The slice coverage of the surface coil used for this study was limited, and had low signal to noise, or no coverage, in most of the caudal regions where the ventral tegmental area (VTA) is located. Thus, the midbrain areas, including the VTA, were not analyzed in this study. In future experiments for further validation of KB220Z, it will be crucial to examine the VTA, the main source of dopamine inputs to the NAc. Additional animal experiments including self-administration experiments with various abusable drugs, like cocaine, alcohol, heroin and nicotine using a 4-channel phased array coil system for rat (Rapid MRI, Columbus, OH) improved signal coverage will help in the interpretation of these results.

It is well–known that natural reward and drugs of abuse like opioids converge on the mesolimbic pathway and activate common mechanism of neural plasticity in the nucleus accumbens. Pitchers et al. showed an endogenous opioid–induced neuroplasticity of dopaminergic neurons in the VTA that influenced natural and opiate (morphine) reward [87]. This finding is of interest because the D-phenylalanine present in KB220Z complex is known to act as an enkephalinase inhibitor [88] and may induce recruitment of dopamine-containing neurons especially in carriers of the Dopamine Receptor (DR) D2 A1 allele. Carriers of the DRD2 A1 allele have 30–40 percent fewer D2 receptors than carriers of the DRD2 A2 allele [89]. In future studies, we intend to characterize the neuronal type, as shown to be recruited in the present experiment, following KB220Z administration. Furthermore, the potential of the proliferation of dopamine D2 receptors following KB220Z administration in these animal experiments has not been evaluated. However, human studies are planned, especially in DRD2 A1 carriers, to determine the number of D2 receptors, utilizing PET scans pre and post KB220Z administered over a 30 day period.

Finally, being cognizant of the current clinical utility of MAT and the pros and cons of these valuable FDA approved pharmaceuticals [2, 9, 90–93] caution by the treatment community, especially in long-term utilization is advised and requires further study.

### Achieving better treatment outcomes

Better treatment outcomes require an understanding that the maintenance of steady “dopaminergic homeostasis” [94–96] is essential for achieving pleasure and satisfaction from ordinary daily activities, and for relieving stress. Untreated impairments in the homeostatic balance of the dopamine signaling can facilitate aberrant substance–related disorders and process addictions [97] elucidated in prior publications as RDS [98, 99]. As has been suggested previously, activation rather than blocking mesolimbic dopaminergic reward circuitry in the long-term treatment of RDS as proposed here is the preferred modality [100]. Although, the acute treatment should consist of preferential blocking of postsynaptic NAc dopamine receptors (D_1_-D_5_), the long-term mesolimbic activation of the dopaminergic system should involve the release and activation of dopamine at the NAc site.

Importantly, based on Ferenczi et al. [70] our observation of KB220Z induced BOLD activation across select regions of the brain strongly suggests the involvement of dopamine and points to the need for optimal dopamine neuronal midbrain stimulation and a glutaminergic-dopaminergic balance that results in dopamine release in the VTA–NAc. The finding here was that KB220Z treatment significantly increased connectivity with regions such as NAc, anterior cingulate cortex, prelimbic and infralimbic structures. From the presented data “seed” analysis using NAc does not show significant FC with VTA area (Fig 3A), not even after KB220Z treatment, perhaps due to lack of slice coverage in this study. There is, however, evidence of selective recruitment of neurons into additional brain structures such as the hippocampus, anterior thalamus, and somatosensory regions and this recruitment may be an indication of neuroplasticity.

Although the short-lived, the amino acids in KB220Z metabolize within 4 hours, the discovery that KB220Z increases functional connectivity has enormous implications for treatment of psychiatric conditions like RDS particularly before drug use and addiction, when, as a result of genetic testing, evidence of an addiction predisposition is found [101].

The diagnostic use of MRI to measure rsFC and connectivity volume supports the idea that baseline connectivity and neuroplasticity may be re-established by administration of KB220Z [10] (Fig 7). Most recently Howard et al., eloquently showed that when mice were trained to switch action dynamically at different selected time points, action selection was associated with changes in dopamine signaling in the dorsal striatum and the firing rate of nigrostriatal dopamine neurons. Moreover, dopamine signaling and action selection biases are altered by optogenetic manipulation of dopamine concentration, and genetic deletion of NMDA receptors on dopamine or striatal neurons. The authors suggested that nigrostriatal dopamine has importance for the treatment of substance use disorder due to its crucial role in integrating diverse information for regulating future actions [102]. The idea that baseline connectivity and neuroplasticity may be re-established during addiction recovery requires further investigation and if confirmed, may make it possible to redeem joy in the lives of those who are afflicted by RDS.

## Supporting information

S1 Fig Atlas registration of anatomical and functional scan. **The first two rows** are representative of Raw EPI and Fast Spin Echo images prior to processing. **The second two** rows are representative assessment of the quality of functional to atlas registration. **In the last three rows** processed functional and anatomical scans were registered to atlas. Each scan was assessed for the quality of alignment of the anatomical scan with the atlas, and consequently of the functional scan transformed to atlas space. Internal brain landmarks such as the corpus callosum, ventricles, internal capsule and others were adequately aligned. Temporal lobe areas had misalignments primarily due to distortions in air-tissue interface regions. These areas were not included in the analysis.
(TIF)

S2 Fig Seed based resting state functional connectivity in individual placebo control rats. Connectivity maps are threshold at z ≥ 0.3. In each panel the top maps represent connectivity with the left and right accumbens and the bottom left and right dorsolateral striatum. Green circles highlight voxels showing bilateral connectivity and arrow points to seed region.
(TIF)

S3 Fig Correlation across different brain regions may be associated with artifacts, especially motor artifacts during scanning. These were controlled and monitored. Anesthetized animals (1–1.5% isofluorane) maintained a breathing rate between 50–70 beats per minute and core body temperature kept at 37–38°C. Baseline images before oral delivery of KB220Z or placebo were removed from the analysis of cross-correlations and the remaining 10 minutes worth of images used for the analysis. Scans were skull stripped, and motion correction applied to realign images to the first in the time series. Further alignment to the high-resolution MRI atlas of the rat brain was carried out on spatially smoothed images (Gaussian FWHM 1.1mm). Images were removed if motion exceeded 0.05mm in the x-y plane (read and phase directions). Minimal movement was observed to occur along the slice direction (z-plane). The figure shows the minimal motion artifact in the top row plots corresponding to animals included in the study for placebo and KB220Z groups. The bottom plots show the animals that were removed due to excess movement artifact.
(TIFF)

S1 Table Medication Assisted Treatment (MAT). (DOCX)

S2 Table Animal studies and clinical trials of KB220 variants. (DOCX)
