# Supplementary figures and images for: Enhanced functional connectivity and volume between cognitive and reward centers of naïve rodent brain produced by pro-dopaminergic agent KB220Z
Source: PLoS One. 2017 Apr 26;12(4):e0174774. doi: 10.1371/journal.pone.0174774 (PMC5405923; doi:10.1371/journal.pone.0174774)

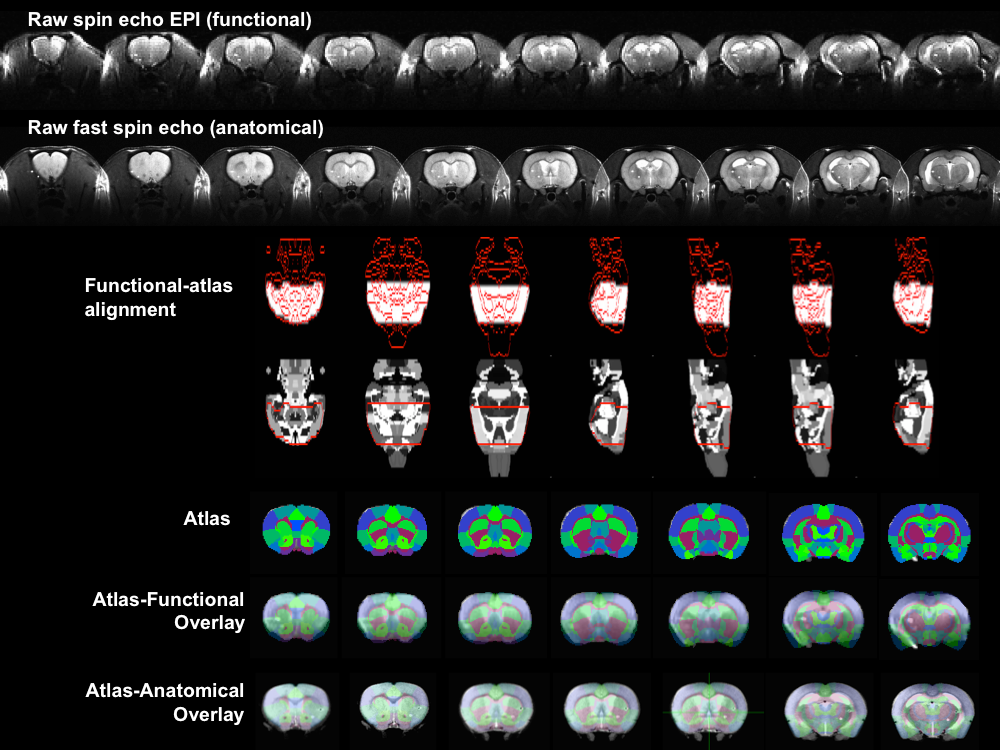

Supplement: S1 Fig — The first two rows are representative of Raw EPI and Fast Spin Echo images prior to processing. The second two rows are representative assessment of the quality of functional to atlas registration. In the last three rows processed functional and anatomical scans were registered to atlas. Each scan was assessed for the quality of alignment of the anatomical scan with the atlas, and consequently of the functional scan transformed to atlas space. Internal brain landmarks such as the corpus callosum, ventricles, internal capsule and others were adequately aligned. Temporal lobe areas had misalignments primarily due to distortions in air-tissue interface regions. These areas were not included in the analysis. (TIF) [file pone.0174774.s001.tif]

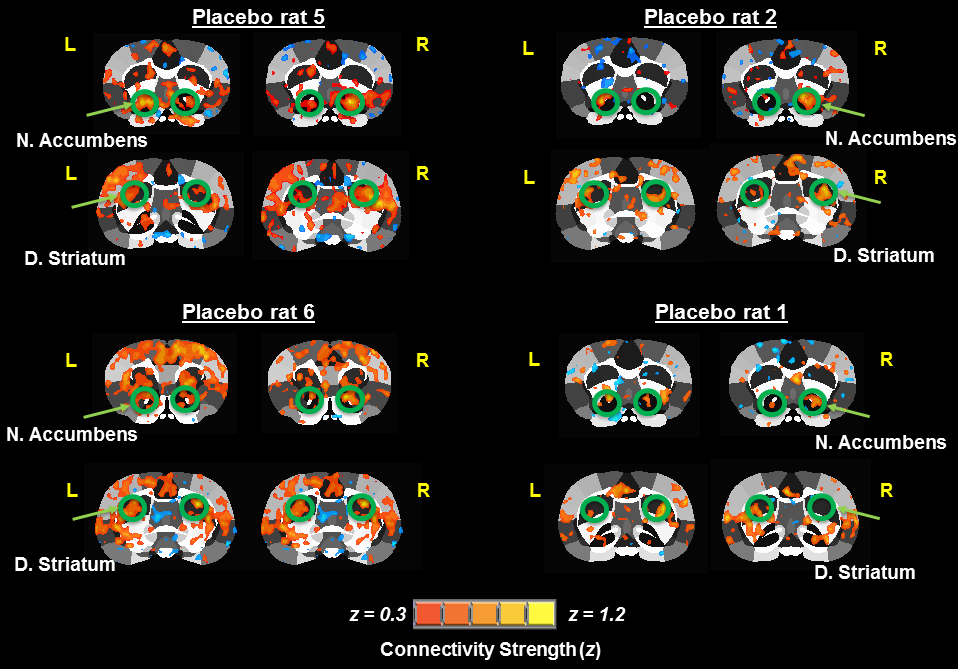

Supplement: S2 Fig — Connectivity maps are threshold at z ≥ 0.3. In each panel the top maps represent connectivity with the left and right accumbens and the bottom left and right dorsolateral striatum. Green circles highlight voxels showing bilateral connectivity and arrow points to seed region. (TIF) [file pone.0174774.s002.tif]

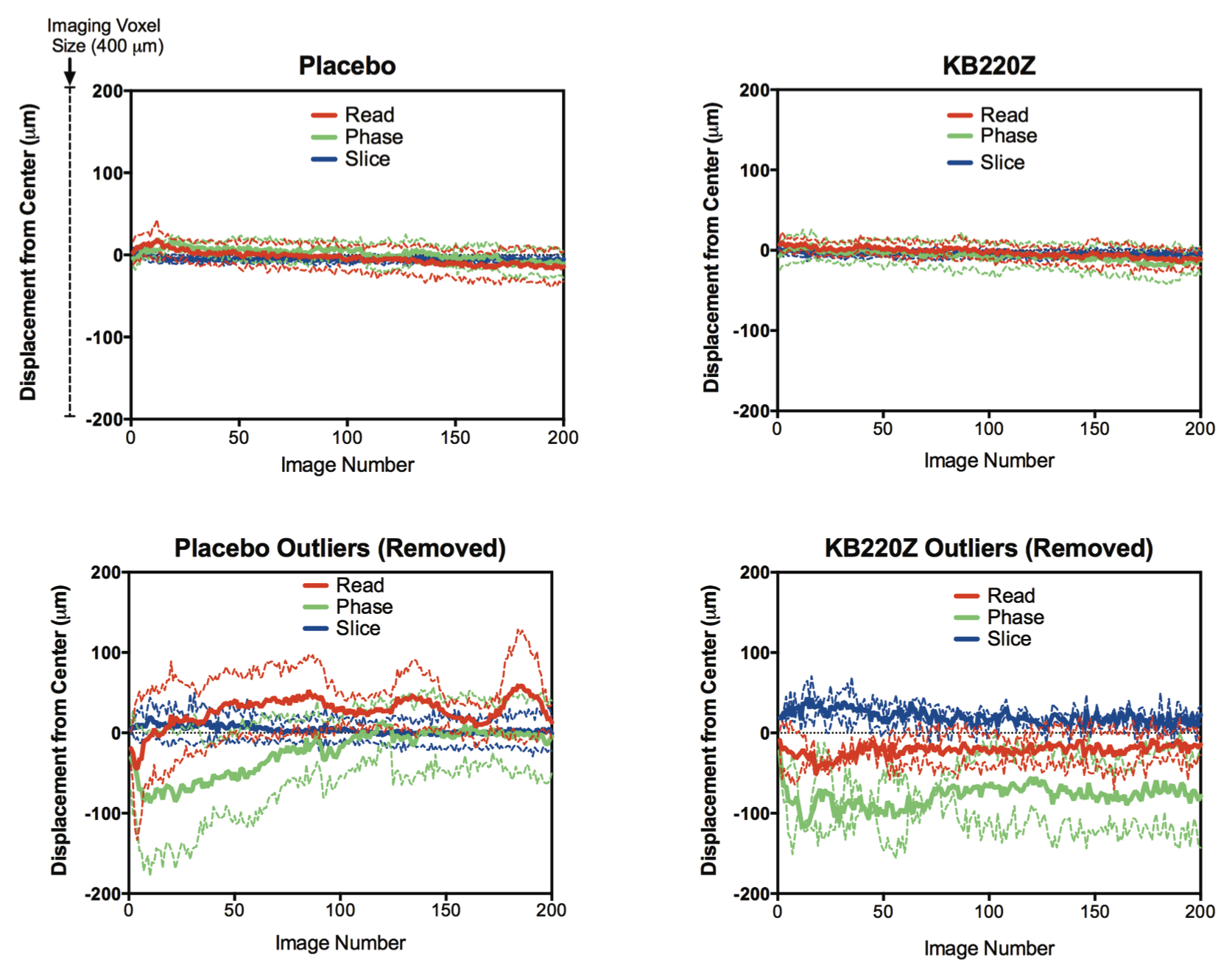

Supplement: S3 Fig — These were controlled and monitored. Anesthetized animals (1–1.5% isofluorane) maintained a breathing rate between 50–70 beats per minute and core body temperature kept at 37–38°C. Baseline images before oral delivery of KB220Z or placebo were removed from the analysis of cross-correlations and the remaining 10 minutes worth of images used for the analysis. Scans were skull stripped, and motion correction applied to realign images to the first in the time series. Further alignment to the high-resolution MRI atlas of the rat brain was carried out on spatially smoothed images (Gaussian FWHM 1.1mm). Images were removed if motion exceeded 0.05mm in the x-y plane (read and phase directions). Minimal movement was observed to occur along the slice direction (z-plane). The figure shows the minimal motion artifact in the top row plots corresponding to animals included in the study for placebo and KB220Z groups. The bottom plots show the animals that were removed due to excess movement artifact. (TIFF) [file pone.0174774.s003.tiff]
