# Supplementary material for: Enhanced functional connectivity and volume between cognitive and reward centers of naïve rodent brain produced by pro-dopaminergic agent KB220Z
Source: PLoS One. 2017 Apr 26;12(4):e0174774. doi: 10.1371/journal.pone.0174774 (PMC5405923; doi:10.1371/journal.pone.0174774)
Supplement: S1 Table — (DOCX) [file pone.0174774.s004.docx]

**S1 Table. United States Federal Drug Authority (FDA) approved pharmaceutical agents:**

**Medication Assisted Treatment (MAT) of Substance Use Disorders**

| **Drug** | **Company** | **Purpose** | **Approval Date** |
| --- | --- | --- | --- |
| Zubsolv®. Zubsolv® (buprenorphine and naloxone) | Orexo AB | treatment of opioid dependence | July 2013 |
| Vivitrol® extended release naltrexone | Alkermes | prevention of relapse to opioid dependence | October 2010 |
| Vivitrol® Naltrexone | Alkermes | treatment of alcohol dependence | April 2006 |
| **CHANTIX**® (varenicline), | Pfizer | treatment of nicotine addiction | May 2006 |
| acamprosate calcium | Campral | treatment of alcoholism | 2004 |
| Suboxone® (buprenorphine/naloxone) Subutex® (buprenorphine) | Reckitt Benckiser | treatment of opiate dependence | October 2002 |
| \|  \| Nicoderm CQ ® \| Nicorette® \| \| --- \| --- \| --- \| | GlaxoSmithKline | for smoking cessation | May 1997 |
| Naltrexone Hydrochloride oral tablets | Dupont | tablet form (50mg taken daily) for the treatment of alcoholism | 1994 |
| Antabuse® (disulfiram) | Odyssey Pharmaceuticals | Treatment of alcohol dependence | 1951 |
