# Supplementary material for: Enhanced functional connectivity and volume between cognitive and reward centers of naïve rodent brain produced by pro-dopaminergic agent KB220Z
Source: PLoS One. 2017 Apr 26;12(4):e0174774. doi: 10.1371/journal.pone.0174774 (PMC5405923; doi:10.1371/journal.pone.0174774)
Supplement: S2 Table — (DOCX) [file pone.0174774.s005.docx]

| **Phase 1** | | |
| --- | --- | --- |
| **Year** | **Reference** | **Key points** |
| 1973 | Blum K, Calhoun W, Merritt J, et al., L-DOPA: effect on ethanol narcosis and brain biogenic amines in mice. *Nature*. 242: 407-409. | Increased brain L-DOPA increases brain dopamine in mice and causes inebriated mice to sleep. Dopamine, 1-tryptophan and alcohol work similarly in the brain. |
| 1974 | Blum K, Wallace JE, Calhoun W, et al., Ethanol narcosis in mice: serotonergic involvement. *Experientia* 30:1053-1054. | When mice were given alcohol and 1-tryptophan or saline the mice given 1-tryptophan went to sleep. The mice given saline did not. 1-tryptophan and alcohol work similarly in the brain. |
| 1987 | Blum K, Wallace JE, Trachtenberg MC, et al., Enkephalinase inhibition: Regulation of ethanol intake in mice. *Alcohol*: 4; 449-456. | Mice genetically predisposed to like alcohol have a measured deficiency in enkephalin. D-phenylalanine and hydrocinnamic acid are substances known to stop the breakdown of enkephalin in the brain -the amount of enkephalin available in the brain increases. When the amount of enkephalin available in the brain increases both voluntary and forced intake of alcohol decreases. D-phenylalanine is one of the ingredients in NAAT. |
| **Phase 2** | | |
| **Year** | **Reference** | **Key points** |
| 1988 | [Blum K](http://www.ncbi.nlm.nih.gov/pubmed?term=%22Blum%20K%22%5BAuthor%5D), [Trachtenberg MC](http://www.ncbi.nlm.nih.gov/pubmed?term=%22Trachtenberg%20MC%22%5BAuthor%5D), [Elliott CE](http://www.ncbi.nlm.nih.gov/pubmed?term=%22Elliott%20CE%22%5BAuthor%5D), et al., Improvement of inpatient treatment of the alcoholic as a function of neurotransmitter restoration: a pilot study. *The International journal of the addictions* 23: 991-8. | First small clinical trial of SAAVE (precursor amino acid loading and enkephalinase inhibition -earliest version of NAAT). Designed to elevate levels of enkephalin(s), serotonin, catecholamines, and GABA, thought to be deficient in alcoholics. Compared to controls those who took SAAVE had lower building up to drink score, required no PRN benzodiazepines, ceased having tremors 24 hours earlier, and had less depression. |
|  | [Blum K](http://www.ncbi.nlm.nih.gov/pubmed?term=Blum%20K%5bAuthor%5d&cauthor=true&cauthor_uid=3072969), [Trachtenberg MC](http://www.ncbi.nlm.nih.gov/pubmed?term=Trachtenberg%20MC%5bAuthor%5d&cauthor=true&cauthor_uid=3072969), [Elliott CE](http://www.ncbi.nlm.nih.gov/pubmed?term=Elliott%20CE%5bAuthor%5d&cauthor=true&cauthor_uid=3072969), et al., Enkephalinase inhibition and precursor amino acid loading improves inpatient treatment of alcohol and polydrug abusers: double-blind placebo-controlled study of the nutritional adjunct SAAVE. [*Alcohol.*](http://www.ncbi.nlm.nih.gov/pubmed/3072969) 5(6): 481-93. | Double blind placebo controlled clinical trial of SAAVE of 62 people with Substance Use Disorder (SUD). Results reduced stress as measured by skin conductance, improved Physical and BESS (behavioral, emotional, social and spiritual) Scores, and had a six-fold decrease in leaving Against Medical Advice (AMA) rates. |
|  | Blum K, Allison D, Trachtenberg MC, et al., Reduction of both drug hunger and withdrawal against advice rate of cocaine abusers in a 30 day inpatient treatment program by the neuronutrient Tropamine. *Current Therapeutic Research* 43: 1204-1214. | Comparison of the effects of Tropamine [T] – (amino acid and vitamin supplement), SAAVE [S]-(a neuronutrient supplement) and no supplement [C] on a group of cocaine abusers in a 30 day hospital treatment program. AMA rate [C] 37.5%, [S] 26.6%, and [T] 4.2 %. Tropamine decreased the AMA rate by significant reduction of drug hunger. |
| 1990 | Brown RJ, Blum K, Trachtenberg, MC, Neurodynamics of relapse prevention: a neuronutrient approach to outpatient DUI offenders. *Psychoactive Drugs* 22: 173-187. | Relapse prevention using neuronutrients SAAVE and Tropamine in DUI offenders; either alcohol or cocaine. Reduced relapse rates and enhanced recovery in 10 week outpatient setting. After 10 months recovery rate was SAAVE 73% and Tropamine 53%. |
|  | Blum K, Trachtenberg MC, Cook DW. Neuronutrient effects on weight loss in carbohydrate bingers; an open clinical trial., *Curr Ther Res.*48: 217-233. | Examine the effects of PCAL-103 (NAAT) on compulsive eating and weight loss in 27 outpatients attending a supervised diet-controlled treatment program. The PCAL-103 average weight loss was 26.96 lbs vs. 10.2 lbs in the control group. Relapse 18.2% in the PCAL-103 group vs 81.8% in the control group. |
| 1996 | Cold JA, NeuRecover-SATM in the Treatment of Cocaine Withdrawal and Craving: A Pilot Study. *Clinical Drug Investigation*. 12(1):1-7, | Small preliminary study of efficacy of NeuRecover-SATM (formerly Tropamine) in the treatment of cocaine withdrawal and craving. Cocaine craving decreased significantly in the NeuRecover-SATM group. |
| 1997 | DeFrance JF, Hymel C, Trachtenberg MC, et al., Enhancement of attention processing by Kantroll in healthy humans: a pilot study. *Clinical Electroencephalography* 28: 68-75. | Cognitive processing speeds in normal young adult volunteers were measured before and after 28-30 days of supplementation with a combination of amino acids (NAAT), vitamins and minerals. Cognitive processing speeds were enhanced by a statistically significant amplitude of the P300 component of the Event Related Potentials (ERPs). FOCUS IMPROVED |
|  | Blum K, Cull JG, Chen TJH, et al., Clinical evidence for effectiveness of Phencal™ in maintaining weight loss in an open-label, controlled, 2-year study. *Current Therapeutic Research* 55(10) 745-763. | Of 247 Outpatients in a very-low-calorie fasting program 130 who were having difficulty attaining their desired weight or maintaining their desired weight constituted the experimental group who took PhenCal™ and the rest 117 took vitamins 117 were the control group. The PhenCal™ group compared to the control lost twice as much weight, regained 14.7% of the weight while the control group regained 41.7%, decrease in food cravings for females 70% and males 63%, and decreased in binge eating for females 66% and males 41%. |
| 2001 | Ross J. Amino-acid precursor and enkephalinase inhibition therapy: evidence for effectiveness in treatment of “Reward Deficiency Syndrome (RDS) with particular emphasis on eating disorders. Mol Psychiatry. Feb; 6(1 Suppl 1):S1-8. | Preliminary evaluation of six randomly selected former eating disordered female clients (three were also chemically dependent), contacted at 9 months and 3 years of treatment with amino-acid precursor and enkephalinase inhibition therapy. All 6 reported initial benefit, one relapsed at 6 months the other 5 all sustained, and in some cases exceeded expectations. 98% of 100 patients similarly treated and evaluated reported significant improvement in both mood and reduced substance craving. |
| 2004 | Chen TJ; Blum K, Payte, JT, et al., Narcotic antagonists in drug dependence: pilot study showing enhancement of compliance with SYN-10, amino-acid precursors and enkephalinase inhibition therapy. *Medical Hypotheses* 63 (3): 538-48. | A combination of Trexan (a narcotic antagonist) and amino-acids was use to detoxify either methadone or heroin addicts. Results were dramatic in terms of significantly enhancing compliance to continue taking Trexan. Trexan alone for rapid detoxification the average number of days of compliance calculated on 1000 patients is 37 days. 12 subjects tested, receiving both the Trexan and amino-acid therapy taking the combination for an average of 262 days. Suggests coupling amino-acid therapy and enkephalinase inhibition, while blocking the delta-receptors with a pure narcotic antagonist as a novel method to induce rapid detox in chronic methadone patients and prevent relapse, and testing this hypothesis with the sublingual combination of the partial opiate mu receptor agonist buprenorphine. |
| 2006 | Blum K, Chen TJ, Meshkin B, et al. Reward deficiency syndrome in obesity: a preliminary cross-sectional trial with a Genotrim variant. *Adv Ther*. 2006 Nov-Dec;23(6):1040-51. | Consumption of large quantities of alcohol or carbohydrates (carbohydrate bingeing) stimulates production and usage of dopamine within the brain. Obesity is due to the need to make up for inadequate dopaminergic activity in the reward center of the brain. This has been called reward deficiency syndrome (RDS) used to categorize such genetic biologic influences on behavior. RDS must be addressed at the same time as behavioral modifications are implemented to adequately treat obese patients. In this small observational trial; 24 individuals completed a survey on which they documented 15 categories of benefit during their experience with a GenoTrim a NAAT formulation customized to DNA. Statistical analysis of the survey results demonstrated that stress reduction lead to improved sleep, enhanced energy, and improved focus and performance, reduced appetite, loss of unwanted weight, decreased body inches, and enhanced well-being. |
| 2007 | Chen TJ, Blum K, Waite RL, et al. Gene \Narcotic Attenuation Program attenuates substance use disorder, a clinical subtype of reward deficiency syndrome. *Advances in Therapy* 24: 402-414. | 1-year prospective study that evaluated the effects of taking Haveos (Synaptamine) on 61 compliant patients in a comprehensive outpatient clinical program. Results after 12 weeks include significant decrease in craving. Results after 1 year include building up to relapse scores and ability to refrain from drug-seeking behavior both significantly improved. The dropout rate for alcohol users 7% and psychostimulant users 73% |
|  | Blum K, Chen TJH, Downs BW, et al. Synaptamine (SG8839), _TM_ An Amino-Acid Enkephalinase Inhibition Nutraceutical Improves Recovery of Alcoholics, A Subtype of Reward Deficiency Syndrome (RDS). *Trends in Applied Sciences Research* 2 (3): 132-138. | In an open clinical study Amino-Acid Enkephalinase Inhibition Nutraceutical improved symptomatology of 600 recovering Alcoholics. Emotional and behavioral recovery scores significantly improved after administration of oral and intravenous Synaptamine. Mean reductions for craving, depression, anxiety, anger, fatigue, lack of energy and crisis were all significantly greater than 50% (p<0.001). |
|  | Chen TJH , Blum K, Kaats G, et al. Chromium Picolinate (Crp) A putative Anti-Obesity Nutrient Induces Changes In Body Composition As Function Of The Taq1 Dopamine D2 Receptor Gene*. Gene Ther Molboil* 11; 161-170. | Chromium Picolinate (CrP) was tested against placebo in groups of obese patients tested for the Taq1 Dopamine D2 Receptor Gene. In carriers of the DRD2 A2 genotype weight loss and other changes in body composition were significant. They were not significant for patients with the A1/A1 or A1/A2 allele. These results suggest that the dopaminergic system, specifically the density of the D2 receptors, confers a significant differential therapeutic effect of CrP in terms of weight loss and change in body fat. |
|  | Blum K, Chen TJH, Williams L, et al. A short term pilot open label study to evaluate efficacy and safety of LG839, a customized DNA directed nutraceutical in obesity: Exploring Nutrigenomics. *Gene Therapy and Molecular Biology* Vol 12, page 371-382. | Preliminary investigational study of evaluate the impact of polymorphisms of five candidate genes on treatment for obesity with NAAT. The formula for each patient was customized based on their genetic results. |
| 2008 | Blum K, Chen AL, Chen TJ, et al., LG839: anti-obesity effects and polymorphic gene correlates of reward deficiency syndrome. *Advances in Therapy* 25 (9): 894-913. | A novel experimental DNA-customized nutraceutical, LG839. Polymorphic correlates were obtained for a number of genes (LEP, PPAR-gamma2, MTHFR, 5-HT2A, and DRD2 genes) with positive clinical parameters tested in this study. Significant results were observed for weight loss, sugar craving reduction, appetite suppression, snack reduction, reduction of late night eating, increased energy etc. Only the DRD2 gene polymorphism (A1 allele) had a significant Pearson correlation with days on treatment. |
|  | Blum K, Chen TJH, Chen ALC, et al., Dopamine D2 Receptor Taq A1 allele predicts treatment compliance of LG839 in a subset analysis of pilot study in the Netherlands. *Gene Therapy Molecular Biology* 12, 129-140. | Hypothesized that genotyping certain known candidate genes would provide DNA-individualized customized nutraceuticals that may have significant influence on body re-composition by countering various genetic traits. Genotyped for the dopamine D2 receptor (DRD2), methylenetetrahydrofolate reductase (MTHFR), serotonin receptor (5-HT2a), Peroxisome Proliferator Activated Receptor gamma (PPAR-γ), and Leptin (OB) genes. Systematically evaluated the impact of polymorphisms of these five candidate genes as important targets for the development of a DNA-customized nutraceutical LG839 [dl-phenylalanine, chromium, l-tyrosine other select amino-acids and adaptogens] to combat obesity with special emphasis on body recomposition as measured by Body Mass Index (BMI). In the 41 day period we found a trend in weight loss whereby 71.4% of subjects lost weight. |
| 2009 | Blum K, Chen ALC, Chen TJH, et al., Putative targeting of Dopamine D2 receptor function in Reward Deficiency Syndrome (RDS) by Synaptamine Complex™ Variant (KB220): Clinical trial showing anti-anxiety effects*. Gene Therapy Molecular Biology* 13, 214-230. | Brain dopamine has been implicated as the so-called “anti-stress molecule.” The present study investigated anti-anxiety effects of Synaptamine Complex [KB220], a dopaminergic activator, in a randomized double-blind placebo controlled study in alcoholics and in polydrug abusers attending an in-patient chemical dependency program. Patients receiving Synaptamine Complex [KB220] had a significantly reduced stress response as measured by SCL, compared to patients receiving placebo. |
| 2010 | Braverman ER, Braverman D, Acrui V, et al., Targeting Noradrenergic and dopaminergic Mechanistic Sites, Hormonal Deficiency Repletion Therapy and Exercise: A case report. *The American Journal of Bariatric Medicine*. 25 (2)18-28, 2010. | Case study evaluating sustained weight loss with Synaptamine complex in conjunction with Diethypropion (Tenuate®), hormonal repletion therapy; use of the Rainbow Diet® and light exercise. After one year, the 58 year old patient's BMT decreased from 32 to 25.4kg/m2 representing a 6.9kg/m2 reduction. His body fat composition decreased from 36.91% to 17.8% as measured by the Hologic DEXA scanner. |
|  | Miller DK, Bowirrat A, Manka M, et al., Acute intravenous synaptamine complex variant KB220™ "normalizes" neurological dysregulation in patients during protracted abstinence from alcohol and opiates as observed using quantitative electroencephalographic and genetic analysis for reward polymorphisms: part 1, pilot study with 2 case reports. *Postgrad Med*. Nov; 122(6):188-213. | Intravenous Synaptamine complex in protracted abstinence from alcohol and opiates analyzed by qEEG. Report that the qEEGs of an alcoholic and a heroin abuser with existing abnormalities (i.e., widespread theta and widespread alpha activity, respectively) during protracted abstinence are significantly normalized by the administration of 1 intravenous dose of Synaptamine Complex Variant KB220 |
|  | Blum K, Chen TJ, Morse S, et al., Overcoming qEEG abnormalities and reward gene deficits during protracted abstinence in male psychostimulant and polydrug abusers utilizing putative dopamine D₂ agonist therapy: part 2. *Postgrad. Med.* Nov; 122(6):214-26. | Protracted Abstinence in Psychostimulant abusers. qEEG analysis in DRD2 A1 allele carriers. Compared to placebo -Synaptose Complex KB220Z induced positive regulation of the dysregulated electrical activity of the brain in these addicts. |
| 2011 | Blum K, Stice E, Liu Y, et al., “Dopamine Resistance” in brain reward circuitry as a function of DRD2 gene receptor polymorphisms in RDS: Synaptamine complex variant (KB220) induced “Dopamine Sensitivity” and enhancement of happiness. *XIX World Congress of Psychiatric Genetics*, September 10-14th. Washington DC. | Synaptamine Complex Variant [KB220] as an activator of the meso-limbic system and administration significantly reduces or “normalizes” aberrant electrophysiological parameters of the reward circuitry site. Based on our QEEG studies presented herein we cautiously suggest that long-term activation of dopaminergic receptors (i.e., DRD2 receptors) will result in proliferation of D2 receptors leading to enhanced "dopamine sensitivity" and an increased sense of happiness. Oral KB220 showed an increase of Alpha activity and an increase low Beta activity similar to 10-20 sessions with Neurofeedback. |
| 2012 | Chen D, Liu Y, He W, et al., Neurotransmitter-precursor-supplement Intervention for Detoxified Heroin Addicts. *Huazhong University of Science and Technology and Springer-Verlag Berlin Heidelberg [Med Sci* 32(3):422-427,2012 | This study examined the effects of combined administration of tyrosine, lecithin, L-glutamine and L-5-hydroxytryptophan (5-HTP) on heroin withdrawal syndromes and mental symptoms in detoxified heroin addicts. The results showed that the insomnia and withdrawal scores were significantly improved over time in participants in the intervention group as compared with those in the control group. A greater reduction in tension-anxiety, depression-dejection, anger-hostility, fatigue-inertia and total mood disturbance, and a greater increase in their vigor-activity symptoms were found at day 6 in the intervention group than in the control placebo group |
|  | Miller M, Chen ALC, Stokes SD, et al., Early Intervention of Intravenous KB220IV- Neuroadaptagen Amino-Acid Therapy (NAAT)™ Improves Behavioral Outcomes in a Residential Addiction Treatment Program: A Pilot Study. *Journal of Psychoactive Drugs* (in press December issue 2012). | In 129 patients a combination of IV and oral NAAR therapy (generic KB220) were assessed for Chronic Abstinence Symptom Severity (CASS) Scale over a 30 day period. Three scales were constructed based on this factor analysis: Emotion, Somatic, and Cognitive. All three scales showed significant improvement (P=0.00001) from pre-to post –treatments: t=19.1 for Emption, t=16.1 for Somatic, and t= 14.9 for impaired cognitive. A two year follow-up in a subset of 23 patients showed: 21(91%) were sober at 6 months with 19(82%) having no relapse; 19 (82% were sober at one year with 18 (78%) having no relapse; 21(91%) were sober at two-years post-treatment with 16(70%) having no relapse. Note: these results of cause do not reflect any other recovery skills utilized by the patients including 12 steps program and Fellowship. |
|  | Blum K, Miller M, Miller D, et al., Neurogenetics and Nutrigenomics of Neuro-Nutrient Therapy for Reward Deficiency Syndrome: Clinical Ramifications and Pitfalls. *Nutrients*. 2012 Nov 27. doi: 10.4172/2155-6105.1000139 | New Definition of Addiction by American Society of Addiction Medicine (ASAM) is based on concepts related to Reward Deficiency Syndrome(RDS). Brain Reward Cascade (BRC) Impairment leads to aberrant craving behavior and other behaviors such as Substance Use Disorder (SUD) due to a “hypodopaminergic” trait/state. Any impairment due to either genetics or environmental influences on this cascade will result in a reduced amount of dopamine release in the brain reward site. After over four decades of development, neuro-nutrient therapy has provided important clinical benefits when appropriately utilized. |
| 2013 | Blum K, Oscar-Berman M, Femino J, et al., Withdrawal from Buprenorphine/Naloxone and Maintenance with a Natural Dopaminergic Agonist: A Cautionary Note. *J Addict Res Ther*. 2013 Apr 23;4(2). doi: 10.4172/2155-6105.1000146. | A case study of a 35 year old female in the film industry with a history of chronic pain from reflex sympathetic dystrophy and fibromyalgia. Total monthly prescription costs including supplemental benzodiazepines, hypnotics and stimulants exceeded $50,000. Withdrawal symptoms were carefully documented when she precipitously stopped taking buprenorphine/naloxone. At 432 days post Suboxone® withdrawal the patient is being maintained on KB220Z, has been urine tested and is opioid free. Genotyping data revealed a moderate genetic risk for addiction showing a hypodopaminergic trait. |
| 2015 | McLaughlin T, Blum K, Oscar-Berman M, et al., Putative dopamine agonist (KB220Z) attenuates lucid nightmares in PTSD patients: Role of enhanced brain reward functional connectivity and homeostasis redeeming joy. *J Behav Addict*. 2015 Jun;4(2):106-15. doi: 10.1556/2006.4.2015.008. | Lucid dreams may be associated with psychiatric conditions, including Post-Traumatic Stress Disorder (PTSD) and Reward Deficiency Syndrome-associated diagnoses. We present two cases of dramatic alleviation of terrifying lucid dreams in patients with PTSD. The medication visit notes reveal changes in the frequency, intensity and nature of these dreams after the complex putative dopamine agonist KB220Z was added to the first patient's regimen. The second PTSD patient, who had suffered from lucid nightmares, was administered KB220Z to attenuate methadone withdrawal symptoms and incidentally reported dreams full of happiness and laughter. |
|  | McLaughlin T, Blum K, Oscar-Berman M, et al., Using the Neuroadaptagen KB200z™ to Ameliorate Terrifying, Lucid Nightmares in RDS Patients: the Role of Enhanced, Brain-Reward, Functional Connectivity and Dopaminergic Homeostasis. *J Reward Defic Syndr*. 2015;1(1):24-35. | Lucid dreams could be un-pleasant or terrifying, at least in the context of patients, who also exhibit characteristics of Reward Deficiency Syndrome (RDS) and Posttraumatic Stress Disorder (PTSD). We present eight clinical cases, with known substance abuse, childhood abuse and diagnosed PTSD/RDS. The administration of a putative dopamine agonist, KB200Z™, was associated with the elimination of unpleasant and/or terrifying, lucid dreams in 87.5% of the cases presented, whereas one very heavy cocaine abuser showed a minimal response. These results required the continuous use of this nutraceutical. If these results in a small number of patients are indeed confirmed we may have found a frontline solution to a very perplexing and complicated symptom known as lucid dreams. |
|  | Blum K, Liu Y, Wang W, et al., rsfMRI effects of KB220Z on neural pathways in reward circuitry of abstinent genotyped heroin addicts. *Postgrad Med*. 2015 Mar;127(2):232-41. | Willuhn et al., reported that cocaine use and even non-substance-related addictive behavior increases as dopaminergic function is reduced. Chronic cocaine exposure has been associated with decreases in D2/D3 receptors and was also associated with lower activation of cues in occipital cortex and cerebellum, in a recent PET study by Volkow's et al., KB220Z induced an increase in BOLD activation in caudate-accumbens-dopaminergic pathways compared to placebo following 1-hour acute administration in abstinent heroin addicts. Increased functional connectivity was observed in a putative network that included the dorsal anterior cingulate, medial frontal gyrus, nucleus accumbens, posterior cingulate, occipital cortical areas, and cerebellum. Results suggest a putative anti-craving/anti-relapse role of KB220Z in addiction by direct or indirect dopaminergic interaction. |
